# Structural basis for PoxtA-mediated resistance to phenicol and oxazolidinone antibiotics

**DOI:** 10.1038/s41467-022-29274-9

**Published:** 2022-04-06

**Authors:** Caillan Crowe-McAuliffe, Victoriia Murina, Kathryn Jane Turnbull, Susanne Huch, Marje Kasari, Hiraku Takada, Lilit Nersisyan, Arnfinn Sundsfjord, Kristin Hegstad, Gemma C. Atkinson, Vicent Pelechano, Daniel N. Wilson, Vasili Hauryliuk

**Affiliations:** 1 Institute for Biochemistry and Molecular Biology, University of Hamburg, Martin-Luther-King-Platz 6, 20146 Hamburg, Germany; 2 Laboratory for Molecular Infection Medicine Sweden (MIMS), Umeå University, 90187 Umeå, Sweden; 3 Umeå Centre for Microbial Research (UCMR), Umeå University, 901 87 Umeå, Sweden; 4 Department of Molecular Biology, Umeå University, 90187 Umeå, Sweden; 5 Department of Clinical Microbiology, Rigshospitalet, 2200 Copenhagen, Denmark; 6 SciLifeLab, Department of Microbiology, Tumor and Cell Biology. Karolinska Institutet, 171 65 Solna, Sweden; 7 University of Tartu, Institute of Technology, 50411 Tartu, Estonia; 8 Faculty of Life Sciences, Kyoto Sangyo University, Kamigamo, Motoyama, Kita-ku, Kyoto, 603-8555 Japan; 9 Department of Microbiology and Infection Control, Norwegian National Advisory Unit on Detection of Antimicrobial Resistance, University Hospital of North Norway, Tromsø, Norway; 10 Research Group for Host-Microbe Interactions, Department of Medical Biology, Faculty of Health Sciences, UiT The Arctic University of Norway, NO-9037 Tromsø, Norway; 11 Department of Experimental Medical Science, Lund University, 221 00 Lund, Sweden

**Keywords:** Cryoelectron microscopy, Antimicrobial resistance, Ribosome, Antibiotics

## Abstract

PoxtA and OptrA are ATP binding cassette (ABC) proteins of the F subtype (ABCF). They confer resistance to oxazolidinone and phenicol antibiotics, such as linezolid and chloramphenicol, which stall translating ribosomes when certain amino acids are present at a defined position in the nascent polypeptide chain. These proteins are often encoded on mobile genetic elements, facilitating their rapid spread amongst Gram-positive bacteria, and are thought to confer resistance by binding to the ribosome and dislodging the bound antibiotic. However, the mechanistic basis of this resistance remains unclear. Here we refine the PoxtA spectrum of action, demonstrate alleviation of linezolid-induced context-dependent translational stalling, and present cryo-electron microscopy structures of PoxtA in complex with the *Enterococcus faecalis* 70S ribosome. PoxtA perturbs the CCA-end of the P-site tRNA, causing it to shift by ∼4 Å out of the ribosome, corresponding to a register shift of approximately one amino acid for an attached nascent polypeptide chain. We postulate that the perturbation of the P-site tRNA by PoxtA thereby alters the conformation of the attached nascent chain to disrupt the drug binding site.

## Abstract (web summary)

PoxtA confers resistance to ribosome-targeting oxazolidinone (linezolid) and chloramphenicol antibiotics. Here, Crowe-McAuliffe et al. provide structural insights into how binding of PoxtA to the ribosome indirectly promotes drug dissociation.

## Introduction

Antibiotic resistance (ARE) is a growing threat to the efficacy of our current arsenal of clinically approved antimicrobial agents. The ATP-binding cassette (ABC) family of proteins are well-known for their role as multidrug resistance transporters, which use the energy of ATP hydrolysis to drive the efflux of antibiotics from the bacterial cytoplasm^1,2^. In recent years, it has become clear that ARE ABC proteins that belong to subfamily F (ABCF) are not transporters—and thus do not confer resistance via efflux—but rather act via a direct target protection mechanism^3–10^.

ARE–ABCF proteins confer resistance to a diverse range of antibiotics that inhibit protein synthesis by targeting the large subunit (LSU) of the ribosome. Based on the spectrum of antibiotic resistance that they confer ARE–ABCF proteins fall into three functional groups: (i) those that protect from pleuromutilins, lincosamides and streptogramins A (PLS_A_), (ii) those that protect from macrolides and streptogramin B (MS_B_), and, finally, (iii) those that protect from phenicols and oxazolidinones (PhO)^3,4,7–10^. These functional groups do not map exactly to the phylogenetic tree of ARE–ABCFs, in which seven subclasses (ARE1-7) were originally distinguished, but are rather scattered amongst non-ARE–ABCFs, implying that these resistance factors have arisen multiple times by convergent evolution^6^. Despite the divergence in the spectrum of antibiotic resistance, ARE–ABCFs share a common architecture. ARE–ABCFs are comprised of two ABC nucleotide-binding domains (NBD1 and NBD2) that are separated by a helical linker termed an ARD (antibiotic-resistance domain), and, depending on the species, ARE–ABCFs may also have an additional “Arm” subdomain inserted within NBD1 as well as a C-terminal extension (CTE)^6^. In fact, this architecture is similar to many non-ARE–ABCFs, such as the *E. coli* housekeeping ABCF ATPase EttA, in which the ARD equivalent is shorter and referred to as a P-site tRNA-interaction motif (PtIM)^11,12^, thus making it difficult to judge whether many ABCF proteins are actually resistance determinants or endogenous proteins of mostly unknown function^6^.

Cryo-EM structures of ribosomes in complex with ARE–ABCFs that confer resistance to PLS_A_ (ARE1 VgaA_LC_ and VgaL, ARE2 VmlR, ARE3 LsaA) and MS_B_ (ARE1 MsrE) classes of antibiotics have revealed that these proteins bind within the E-site^13–15^, similar to that reported previously for the housekeeping non-ARE–ABCF EttA^11^. However, in contrast to EttA^11,12^, the longer ARD of the ARE–ABCF proteins distorts the P-site tRNA, allowing the factor to access the peptidyl transferase centre (PTC) on the LSU of the ribosome and dislodge the relevant antibiotics from their binding sites^13–15^. These structures revealed that there is often no steric overlap between the ARD of the ARE–ABCF and the drugs and even when there is a steric overlap, the mutational analysis indicated that it is not strictly required for resistance^13–15^. Collectively, these results support a model where the ARE–ABCFs dislodge the drugs from the PTC by inducing a cascade of conformational changes within the 23S rRNA nucleotides that comprise the drug-binding site^15^. For MsrE, drug release was reported to occur in the presence of a so-called EQ_2_ variant of the protein, which is deficient in ATPase activity^14^, suggesting that ATP hydrolysis is not essential for drug release, but rather is needed for recycling of the factor from the ribosome.

In contrast to the relatively well-studied PLS_A_- and MS_B_-protecting ARE–ABCFs, mechanistic insights into how ARE7 OptrA and ARE8 PoxtA confer resistance to PhO antibiotics are lacking. The first ARE–ABCF from this group to be discovered was OptrA. This factor confers resistance to the phenicols, such as chloramphenicol and florfenicol, as well as the oxazolidinones linezolid and, to a lesser extent, tedizolid^16^. OptrA was originally found on the conjugative plasmid pE349 from *Enterococcus faecalis*^16^, but has subsequently been detected, both plasmid and chromosomal-encoded, across many Gram-positive enterococci, staphylococci and streptococci of human and animal origin^16–22^. At least 69 variants of the *optrA* gene have been reported to date, differing by 1–20 aa substitutions, which corresponds to an amino acid identity of 97.1–99.8% compared to the first reported OptrA sequence^22^. Moreover, evidence for horizontal transfer of OptrA to Gram-negative bacteria, such as *Campylobacter coli*, has also recently been described^23,24^. While oxazolidinones are not clinically effective against Gram-negative pathogens, this raises the concern of possible co-selection of antibiotic co-resistance i.e. selection for simultaneous transfer and spread of several antibiotic-resistance genes encoded by one mobile genetic element. In addition to OptrA, a second ARE–ABCF from this group was detected by bioinformatic analysis of the genome of a methicillin-resistant *Staphylococcus aureus* (MRSA) strain AOUC-0915 isolated from a cystic fibrosis patient at the Careggi University Hospital in Florence, Italy^25^. Expression of the resistance determinant in *S. aureus*, *E. faecalis* and *E. coli* was reported to confer resistance to phenicol-oxazolidinone-tetracycline antibiotics, and was therefore termed PoxtA^25^. To date, PoxtA has been found exclusively in *Enterococcus* and *Staphylococcus* species, most frequently in *E. faecium* isolates of both human and animal origin^22^.

**Fig. 1:**
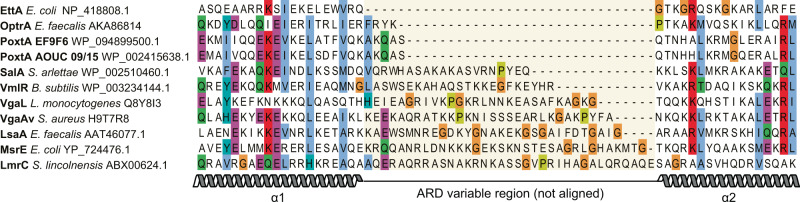
Alignment of the ARDs from diverse bacterial ABCFs. The PoxtA and OptrA ARDs are slightly longer (4–5 amino acids) than the equivalent region in EttA, but significantly shorter than in other ARE–ABCFs. The central region with an orange highlight, which includes the ARD loop and some of the adjacent helices in some proteins, was not aligned but simply ordered by length. Sequences were aligned with MAFFT^70^ and edited by hand to reduce gap placement. Sequence accession numbers in the title are Uniprot or NCBI protein accessions.

In the absence of structures of OptrA and PoxtA on the ribosome, it has remained unclear how these ARE–ABCFs confer antibiotic resistance. Both chloramphenicol and linezolid bind at the PTC and inhibit the elongation phase of protein synthesis^26^. However, their activity is context-specific such that translation arrest is most efficient when the nascent polypeptide chain on the ribosome carries an alanine residue and, to a lesser extent, serine or threonine in its penultimate position^27–29^. Although the ARDs of OptrA and PoxtA are slightly longer (4–5 aa) than the PtIM of non-ARE–ABCFs such as EttA, they are considerably shorter than the ARDs of ARE–ABCFs from other groups, and at least 20 amino acids shorter than other ARE–ABCFs for which structures have been reported (Fig. 1). Thus, assuming OptrA and PoxtA bind similarly to the ribosome as other ARE–ABCFs, the ARDs are unlikely to be able to reach into the PTC to dislodge the drugs from their binding site^8^. Moreover, it is hard to rationalise how PoxtA also confers resistance to tetracycline antibiotics, which bind near the decoding site on the small subunit (SSU), which is located far from the PTC on the LSU^26^.

Here, we systematically characterise the PoxtA and OptrA resistance determinants, revealing that both increase the minimum inhibitory concentrations (MICs) to phenicols, such as chloramphenicol, thiamphenicol and florfenicol, as well as to the oxazolidinone linezolid, but not to macrolides, pleuromutilins, lincomycins and streptogramins that bind adjacent to the PTC within the ribosomal exit tunnel. Moreover, we find no evidence for either PoxtA or OptrA conferring resistance to the non-PTC binding antibiotic tetracycline. Cryo-EM structures of PoxtA on the ribosome reveal that it binds in the E-site and, despite the short ARD, still induces a distortion of the P-site tRNA, leading to retraction of its CCA-end from the PTC. Unlike for other ARE–ABCFs, we observe no conformational rearrangements within the 23S rRNA around the drug-binding sites within the A site of the PTC upon binding of PoxtA to the ribosome. This leads us to propose a model whereby the distortion of the P-site tRNA by PoxtA (and OptrA) reduces the affinity of the drugs for their binding site by altering the context and therefore interaction of the nascent polypeptide chain with respect to the drugs.

## Results

### PoxtA and OptrA confer resistance to phenicols and oxazolidinones, but not tetracyclines

To systematically characterise the antibiotic-resistance profiles of PoxtA and OptrA, representatives of these ARE–ABCF groups were expressed in an *E. faecalis* TX5332 strain where the *lsaA* gene had been disrupted (Δ*lsaA*), and MICs were determined for phenicol (chloramphenicol, thiamphenicol and florfenicol), oxazolidinone (linezolid), macrolide (erythromycin, azithromycin and leucomycin), lincosamide (lincomycin and clindamycin), pleuromutilin (tiamulin, retapamulin), streptogramin A and B (Virginiamycin M1 and S1, respectively) and tetracycline antibiotics (Table 1). We have characterised OptrA E35048 from *E. faecium*^30^, OptrA ST16 from the clinical *E. faecalis* ST16 isolate^21^, PoxtA AOUC-0915 from MRSA^25^ and, finally, PoxtA EF9F6 from a multidrug-resistant ST872 *E. faecium* clinical isolate 9-F-6^31^. As controls, we determined MICs for the *E. faecalis* Δ*lsaA* strain transformed with the empty vector plasmid pCIE_spec_, as well as expressing LsaA, the native genome-encoded ARE–ABCF from *E. faecalis*.

**Table 1:** Antibiotic-resistance spectra of LsaA, OptrA and PoxtA ARE–ABCFs.

	Minimum inhibitory concentration (MIC, μg/mL)					
	pCIE_spec_ vector	LsaA	OptrA ST16	OptrA 35048	PoxtA EF9F6	PoxtA AOUC-0915
Chloramphenicol	2–4	2–4	**8–16**	**4–8**	**4–8**	**4–8**
Thiamphenicol	4	4	**32–64**	**16–32**	**8–16**	**32–64**
Florfenicol	1–2	1–2	**16–32**	**8**	**2–4**	**16–32**
Linezolid	1	1	**8**	**2–4**	**2–4**	**4–8**
Erythromycin	0.5–1	0.5	0.5	0.5	0.5	0.5
Azithromycin	0.5	0.5	0.5	0.5	0.5	0.5
Leucomycin	0.5	0.5	0.5	0.5	0.5	0.5
Lincomycin	0.125	**16–32**	0.125	0.125	0.125	0.125
Clindamycin	0.0156	**16**	0.0156	0.0156	0.0156	0.0156
Tiamulin	0.0625	**128**	0.0156	0.0156	0.031	0.031
Retapamulin	0.0156	**>64**	0.0156	0.0156	0.0156	0.0156
Virginiamycin M1	4	**>128**	4	4	4	4
Virginiamycin S1	8	8	8	8	8	8
Tetracycline	0.25	0.25	0.25	0.25	0.25	0.25

BHI media supplemented with 2 mg/mL kanamycin (to prevent *lsa* revertants), 0.1 mg/mL spectinomycin (to maintain the pCIE_spec_ plasmid), 100 ng/mL of cCF10 peptide (to induce expression of ABCF proteins) as well as increasing concentrations of antibiotics were inoculated with 5 × 10^5^ CFU/mL (≈OD_600_ 0.0005) of *E. faecalis* Δ*lsaA* (*lsa*::Kan) strain TX5332 transformed either with empty pCIE_spec_ plasmid, or with pCIE_spec_ derivatives for expression of ARE–ABCF proteins. After 16–20 h at 37 °C without shaking, the presence or absence of bacterial growth was scored by eye. The MIC values that are higher than the empty vector control are shown in bold. The experiments were performed in triplicates.

In agreement with earlier reports, expression of LsaA confers resistance to PLS_A_ antibiotics, but not PhO, MS_B_ or tetracycline^15,32^. By contrast, cCF10-inducible expression of either OptrA or PoxtA results in a 2- to 16-fold MIC increase for PhO antibiotics, and does not, as expected, result in resistance against either PLS_A_ or MS_B_, as observed in earlier reports^16,25^. While the earlier study^25^ reported a minor protective effect of PoxtA against tetracycline (a twofold increase in MIC for *E. faecalis* and *S. aureus*), in our hands expression of neither of the PoxtA variants resulted in any increase in MIC for this antibiotic. Notably, the lack of effect of PoxtA AOUC-0915 expression on the MIC for the tetracycline antibiotic tigecycline in either *E. faecalis* or *S. aureus*^25^ is consistent with the original *E. faecium* 9-F-6 strain from which we have isolated PoxtA EF9F6 also being susceptible to tigecycline (Supplementary Table 1). Therefore, we concluded that the antibiotic-resistance spectrum of PoxtA is similar, if not identical, to that of OptrA. This similarity appears to be a case of convergent evolution, as there is no phylogenetic support for PoxtA and OptrA being more closely related to each other than to any other ABCF subfamily member (Supplementary Fig. 1). Indeed, OptrA is more closely related to the vertically inherited and probable housekeeping ABCF YdiF of Firmicutes (84% bootstrap support, Supplementary Fig. 1), while the relationship of PoxtA to other subfamilies is unresolved (bootstrap support below 50%). On these grounds, we conclude that PoxtA-like proteins constitute a separate ARE subfamily which we call ARE8.

### PoxtA rescues ribosome stalls associated with linezolid treatment

Extensive in vivo and in vitro studies into the mechanism of action of chloramphenicol and linezolid during *E. coli* protein synthesis have established that both antibiotics act as context-specific inhibitors of translation elongation^27–29^. Specifically, efficient ribosomal stalling by linezolid is strongly stimulated by the presence of alanine as the penultimate amino acid in the polypeptide nascent chain (i.e. the −1 position), while the codon for this amino acid occupies the ribosomal E-site. Dependence of chloramphenicol-mediated stalling on alanine in the −1 position of the nascent chain was previously shown in Gram-positive Firmicute species *Lactobacillus plantarum* and *Bacillus subtilis* using the recently developed bacterial high-throughput 5ʹP sequencing (5Pseq) method^33^. Analogous to the well-established 5Pseq for eukaryotes^34^, the bacterial version of the technique detects ribosome occupancy by generating in vivo footprints of the 5ʹ-most ribosome on the mRNA molecule through specific sequencing of the 5ʹ monophosphorylated intermediates of 5ʹ–3ʹ mRNA degradation^33^.

**Fig. 2:**
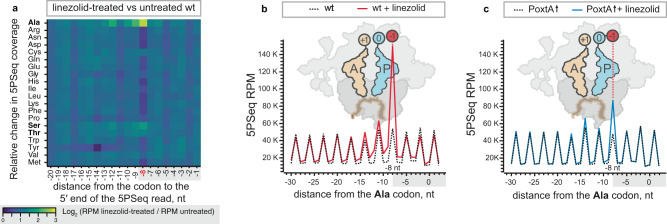
Context-dependent ribosomal stalling induced by linezolid is countered by PoxtA. **a** A heatmap of log_2_-relative change in the 5Pseq coverage of wild-type *E. faecalis* upon linezolid treatment. The distance (in nucleotides, nt) from the 5ʹ of the sequenced mRNA fragments to synonymous codons encoding a specified amino acid is indicated on the *x* axis. The 0 nt position corresponds to the first nucleotide of the codon. Dark blue signifies decreased 5PSeq coverage upon linezolid treatment, yellow signifies increased coverage upon linezolid treatment. A specific increase in the 5Pseq coverage associated with synonymous codons located at –8 nucleotides is indicative of a ribosomal stall with the corresponding amino acid in the −1 position of the nascent chain, as illustrated schematically in panels **b** and **c** (ref. ^33^). **b**, **c** Metagene analysis of 5Pseq libraries aligned to alanine codons. Linezolid-induced ribosomal stalling results in a specific increase in 5Pseq read counts 8 nt upstream of alanine codons, indicating a ribosomal stall with alanine residues in the −1 position (see schematics). Samples were prepared with *E. faecalis* harbouring the empty pCIE_spec_ vector (**a**, **b**) or upon *poxtA* expression (**c**, blue line), with and without linezolid treatment. Exponentially growing cells were treated with 4 μg/mL linezolid (final concentration) 10 min before harvest. All analyses were performed on pooled datasets from three biological replicates.

We used 5Pseq to globally survey the linezolid-induced ribosomal stalls in *E. faecalis* and directly assess the role of PoxtA in overcoming these stalls. 5Pseq analysis of linezolid-treated *E. faecalis* robustly detected increased 5Pseq coverage signal −8 nucleotides upstream of the first (5ʹ) nucleotide of the alanine codon (and to lesser extent at serine codons), which is indicative of ribosomal stalling with these codons in the E-site and their respective amino acids in the −1 position of the nascent chain (Fig. 2a and Supplementary Fig. 2). Importantly, all of the alanine iso-codons display near-identical linezolid-induced stalls, which is indicative of the stalling being defined by the nature of the amino acid, not the codon itself (Supplementary Fig 3). There was no particular enrichment of codons in the A- or P-sites (Fig. 2a). The strong stalling at alanine and weaker stalling at serine and threonine is in good agreement with an *E. coli* ribosome profiling study, indicating that linezolid affects translation similarly in both organisms^27^. PoxtA expression resulted in the reduction of this context-specific stalling signal in response to linezolid (Fig. 2b, c and Supplementary Fig. 2). The rescue effect is highly reproducible between the three biological replicates (Supplementary Fig. 4a–d), and both the linezolid-induced alanine stalling and its rescue by PoxtA expression are statistically significant (Supplementary Fig. 4e).

By contrasting 5PSeq datasets of antibiotic-untreated *E. faecalis* that do not express PoxtA with that of an untreated PoxtA-overexpressing control, we probed whether sampling of the ribosomes by PoxtA in the absence of antibiotics causes ribosomal stalling. Our analyses revealed slightly increased ribosomal stalling at intrinsically slow initiation and termination steps (Supplementary Fig. 5). Importantly, no stalling signal specific for the −1 nascent chain position (corresponding to the ribosomal E-site) was detectable, consistent with linezolid determining the amino acid specificity of antibiotic-induced stalls.

Collectively, our 5Pseq study confirms the generality of the context-specific mechanism of action of linezolid and indicates that PoxtA alleviates the drug-induced stalling at positions with −1 alanine residues.

### Cryo-EM structures of PoxtA–70S complexes

Our attempts to reconstitute OptrA- and PoxtA–70S ribosome complexes in vitro were unsuccessful due to problems obtaining soluble homogenous OptrA and PoxtA proteins. Therefore, we employed an in vivo pull-down approach with strains overexpressing C-terminally FLAG_3_-tagged OptrA and PoxtA proteins, as used recently to generate other ARE–ABCF–ribosome complexes^15^. We expressed both the wild-type ATPase-competent OptrA and PoxtA and EQ_2_ ATPase-deficient variants bearing Glu-to-Gln substitutions in both NBD cassettes. Such EQ_2_ variants have been successfully employed to trap other ABCF proteins on the ribosome because these substitutions allow binding but prevent hydrolysis of ATP by the ABCF^11,13,15,35^. The OptrA-EQ_2_ variant was earlier shown to have a compromised in vitro ATPase activity and unable to confer resistance in vivo^36^. Consequently, the addition of chloramphenicol or linezolid to growth media would block cCF10-driven overexpression of ATPase-deficient OptrA/PoxtA EQ_2_ variants. Therefore, in vivo ABCF–ribosome complex formation and its subsequent immuno-purification were performed in the absence of the antibiotic. Affinity purification via the FLAG_3_ tag was performed in the presence of 0.5 mM ATP from clarified lysates prepared from the *E. faecalis* Δ*lsaA* strain expressing the FLAG_3_-tagged ARE–ABCF, either wild-type or EQ_2_-variant. Analysis of the elution fractions from the purifications indicated that only the *E. faecium* PoxtA(EF9F6)-EQ_2_ factor was bound stably to the ribosome (Supplementary Fig. 6). Furthermore, our attempts with OptrA carrying a single individual EQ substitution (E470Q) were equally unsuccessful. Therefore, we focused on the PotxA(EF9F6)-EQ_2_–70S sample and subjected it to structural analysis using single-particle cryo-EM.

Using a Titan Krios transmission electron microscope with a K2 direct electron detector, we collected 3640 micrographs which, after 2D classification, yielded 140,310 ribosomal particles (Supplementary Fig. 7). In silico sorting revealed that 80% of these particles contained an additional density for PoxtA and/or tRNAs, which after 3D refinement resulted in a cryo-EM map of *E. faecalis* 70S ribosome with an average resolution of 2.4 Å (Supplementary Fig. 8a). Subsequent multibody refinement yielded average resolutions of 2.2 Å and 2.5 Å for the LSU and SSU, respectively (Supplementary Fig. 8b–e). The increase in resolution compared to the previous *E. faecalis* 70S ribosome models at 2.8–2.9 Å^15,37^ is evident from improved quality and features of the cryo-EM density, including visualisation of some rRNA modifications (e.g. N2-methylguanosine), water molecules and hydrated magnesium ions (Supplementary Fig. 9a–f and Supplementary Table 2).

**Fig. 3:**
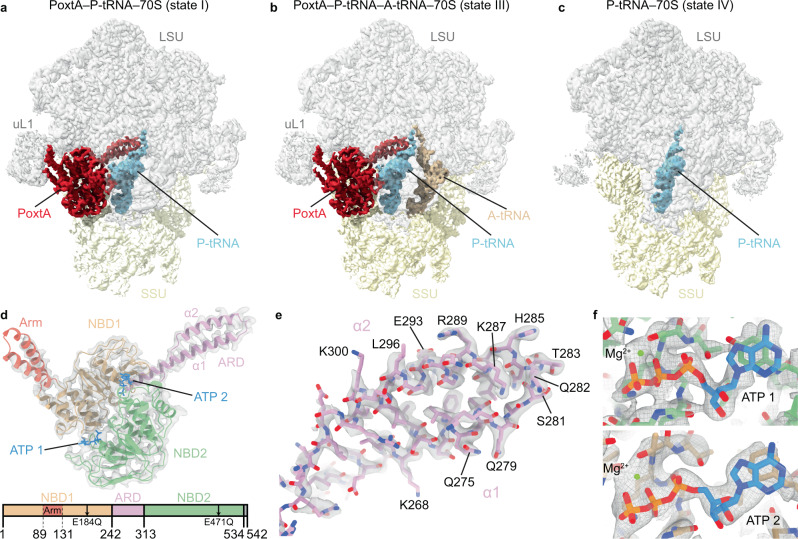
Cryo-EM structures of PoxtA–70S complexes. **a**–**c** Cryo-EM maps with isolated densities for (**a**, **b**) *E. faecium* PoxtA (red) in complex with the *E. faecalis* 70S ribosome and **a** P-tRNA (cyan) or **b** P-tRNA (cyan) and A-tRNA (tan), **c** P-tRNA (cyan) only, with small subunit (SSU, yellow) and large subunit (LSU, grey). **d** Density (grey isosurface) with molecular model of PoxtA from **a** coloured according to domain as represented in the associated schematics: nucleotide-binding domain 1 (NBD1, tan), antibiotic-resistance domain (ARD, pink), nucleotide-binding domain 2 (NBD2, green) and C-terminal extension (CTE, grey, not modelled). α1 and α2 indicate the two α-helices of the ARD interdomain linker. In **d**, the ATP nucleotides are coloured blue. **e** Close view of the ARD tip from state I with sharpened map. **f** Close view of ATPs bound by PoxtA (state I).

Further subsorting of ribosomal particles using a mask focused on the intersubunit space yielded four defined classes, which we refer to as states I–IV (Supplementary Fig. 7). States I and II contained density for PoxtA bound in the E-site and tRNA in the P site, and had average resolutions of 2.9 and 3.0 Å, respectively (Fig. 3a, Supplementary Fig. 10a, b and Supplementary Table 2). State II (not shown in Fig. 3) differed from the state I by only a slight rotation of the SSU relative to the LSU, and in particular the conformation of PoxtA was nearly identical to that of state I (Supplementary Fig. 11). State III was similar to state II, but additionally contained an A-site tRNA and PoxtA differed only slightly in NBD2, close to the SSU (Fig. 3b and Supplementary Fig. 11), whereas state IV contained P-site tRNA only (Fig. 3c), presumably because PoxtA dissociated during sample preparation. States III and IV were also refined, resulting in final reconstructions with average resolutions of 2.9 Å and 3.1 Å, respectively (Supplementary Fig. 10a, b and Supplementary Table 2). The cryo-EM density for PoxtA in states I–III was generally well-resolved (Supplementary Fig. 10c), enabling a reliable model to be built for NBD1, NBD2 and the ARD (Fig. 3d). By contrast, the Arm domain, which interacts with uL1, appeared flexible (Supplementary Fig. 10c) and could only be modelled as a rigid body fit of the two α-helices (Fig. 3d). The best-resolved region of PoxtA was the ARD, consisting of two α-helices (α1 and α2) and the ARD loop (Fig. 3d and Supplementary Fig. 10c), where the majority of sidechains could be modelled unambiguously (Fig. 3e). Additional density located between NBD1 and NBD2 of PoxtA was attributed to two ATP molecules (ATP-1 and ATP-2) and a likely magnesium ion (Fig. 3f), as expected from the use of the ATPase-deficient PoxtA-EQ_2_ variant. As observed for other ribosome-bound ARE–ABCF structures^13–15^, the NBDs of PoxtA adopt a closed conformation, which is also consistent with the inability to hydrolyse ATP. In all states, the anticodon stem loop (ASL) and acceptor arm, including the CCA-end, are well-resolved, whereas the elbow region of the tRNAs exhibits some flexibility (Supplementary Fig. 10d).

In all states, the density for the P-site tRNA is consistent with initiator tRNA^fMet^, indicating that in the absence of chloramphenicol- or linezolid-stalled ribosomes, the PoxtA-EQ_2_ variants bind to the vacant E-site of initiation complexes, as observed previously for other ARE–ABCF-EQ_2_–ribosome complexes prepared by an in vivo pull-down approach^15^. Specific recruitment of PoxtA-EQ_2_ to initiation complexes is likely due to the availability of the E-site rather than due to the specific affinity to initiating ribosomes as such. In state IV, clear density for the fMet moiety attached to the P-site tRNA is evident, whereas state III appears to be a post-peptide bond formation state with a deacylated P-site tRNA and A-site tRNA bearing a dipeptide. In states I and II, which contain PoxtA, but lack A-site tRNA, some density for the fMet moiety on the distorted P-site tRNA is evident but is poorly resolved.

### Interaction of PoxtA with the ribosome and P-site tRNA

**Fig. 4:**
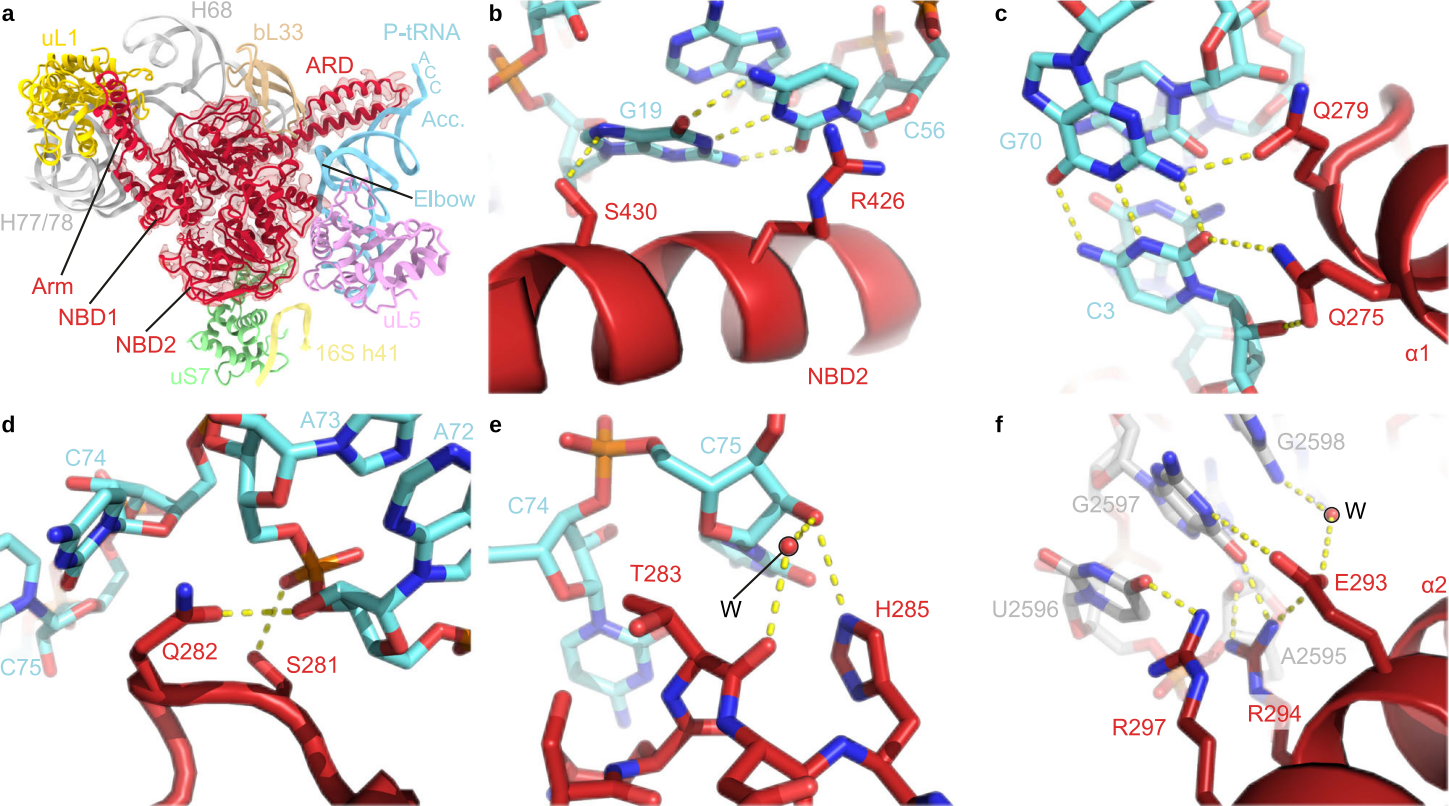
Interactions between PoxtA and the ribosome-P-tRNA complex. **a** Overview of PoxtA interactions with the 23S rRNA (grey), 16S rRNA h41 (yellow), uL1 (gold), uS7 (green), uL5 (pink), bL33 (tan) and the P-tRNA (light blue). The P-tRNA CCA 3ʹ end, acceptor stem (Acc.), and elbow are indicated. **b**–**e** Interactions between the P-tRNA elbow (light blue) and the PoxtA NBD2 (**b**), the P-tRNA acceptor stem (light blue) and the PoxtA ARD (**c**), the ARD and the P-tRNA CCA-end, including a modelled water molecule (labelled W) (**d**, **e**). **f** An interaction between the PoxtA ARD α2 and the 23S rRNA. The high-resolution model from the combined 70S volume was used.

In states I–III, PoxtA is located within the ribosomal E-site (Fig. 4a) and generally binds similarly to that observed for other ARE–ABCF proteins, such as VmlR, MsrE, LsaA, VgaL and VgaA_LC_^13–15^, as well as the non-ARE–ABCF protein EttA^11^. Unlike these ARE–ABCFs that lack an Arm subdomain (or have a short Arm in the case of LsaA)^6^, the Arm subdomain of PoxtA (and OptrA^6^) is prominent, like that of EttA^11,12^, and stabilises an open conformation of the L1 stalk via direct interaction with domain II of uL1 (Fig. 4a). Additional contacts to the 23S rRNA helices H77/H78 of the L1 stalk are evident from the NBD1 of PoxtA, as are interactions for NBD1 with H68 and bL33 on the LSU (Fig. 4a). By contrast, NBD2 of PoxtA spans across the intersubunit interface, establishing interactions with uL5 on the LSU as well as uS7 and h41 on the SSU (Fig. 4A). NBD2 also interacts directly with the elbow region of the P-site tRNA, namely, with the G19-C56 basepair that links the D- and T-loops (Fig. 4a, b). Here, Ser430 of PoxtA is within hydrogen bonding distance of the N7 of G19 and the sidechain of Arg426 of PoxtA stacks upon the nucleobase of C56 of the T-loop (Fig. 4b). However, it is the ARD that makes the most extensive interactions with the P-site tRNA, establishing a complex network of hydrogen bonding interactions with the acceptor arm and CCA-end (Fig. 4a). In particular, two glutamine residues, Gln275 and Gln279, located at the distal end of the α-helix α1 of the ARD insert into the minor groove of the acceptor’s arm where hydrogen bond interactions can form with the C3-G70 basepair (Fig. 4c). Hydrogen bonding is also possible from the ε-amino group of Lys278 and the backbone carbonyl of Gln275 of PoxtA with the phosphate-oxygen of G4 and the ribose-oxygen of C71 of the P-tRNA, respectively. The loop region of the ARD of PoxtA interacts predominantly with the single-stranded CCA-3ʹ end of the P-site tRNA (Fig. 4d). Ser281 is within H-bonding distance to the phosphate-oxygen of A73, whereas the sidechain of Gln282 stacks upon the base of C74 and can interact with the ribose-hydroxyl of A72 (Fig. 4d). C75 of the P-site tRNA is also stabilised by indirect contacts with the backbone carbonyl of Thr283 via a water molecule, as well as a direct H-bond with the sidechain of His285, the first residue of α-helix α2 of the ARD of PoxtA (Fig. 4e). The ARD is stabilised by multiple contacts between residues within α-helix α2 and rRNA nucleotides located in H74 and H93. For example, the sidechains of Arg294 and Arg297 contact nucleotides A2595–G2598 (*E. coli* numbering used throughout) located within the loop of H93 of the 23S rRNA, and Glu293 interacts with G2597 directly as well as G2598 via a putative water molecule (Fig. 4f).

### PoxtA perturbs the position of the CCA-end of the P-site tRNA at the PTC

**Fig. 5:**
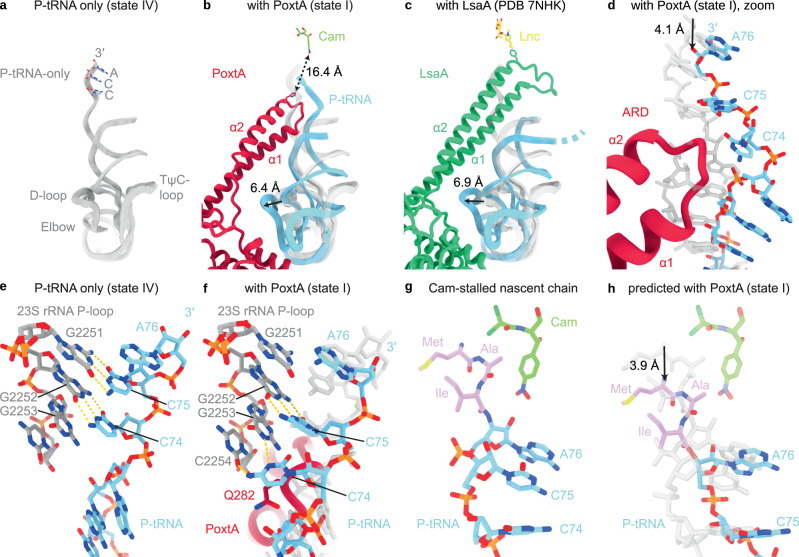
PoxtA modulates the conformation of the P-tRNA. **a**–**c** Comparison of of the P-site tRNA from (**a**) *E. faecalis* 70S–P-tRNA only complex (grey), (**b**) PoxtA–70S complex (state I, PoxtA in red and P-tRNA in light blue) and (**c**) LsaA–70S complex (PDB ID 7NHK^15^, LsaA in green and P-tRNA in light blue). Models of chloramphenicol for PoxtA (PDB ID 6ND5^77^) and lincomycin for LsaA (PDB ID 5HKV)^78^ are superimposed for reference. **d** close-up of (**b**) showing P-tRNA acceptor stem distortion induced by PoxtA binding. **e**, **f** Interaction between the 23S rRNA P-loop (grey) and P-tRNA (light blue) for (**e**) the P-tRNA-only complex (state IV) and **f** with bound PoxtA (state I, state IV P-tRNA is overlayed in transparent grey for comparison). **g**, **h** Indirect modulation of the chloramphenicol binding site by PoxtA. **g** The structure of chloramphenicol (cam) stabilised by a nascent peptide chain with alanine in the −1 position^39^. **h** Modelled shift of the nascent chain shown in **g** induced by PoxtA displacement of the P-tRNA CCA-end. The P-tRNA from **g** is overlaid in transparent grey for comparison.

Despite the short ARD, binding of PoxtA to the E-site nevertheless causes a distortion of the P-site tRNA when compared to the canonical P-site tRNA binding position, such as that observed in state IV (Fig. 5a, b). While the ASL remains fixed in position on the SSU where it decodes the P-site codon of the mRNA, the elbow region shifts towards the E-site by ~6–7 Å, thus bringing it into contact with NBD2 of PoxtA (Fig. 5b). The shift of the elbow region is very similar to that observed for the distorted P-site tRNAs observed on the ribosome in the presence of the other ARE–ABCFs^13–15^ such as LsaA (Fig. 5c). However, in the PoxtA–70S complex, the distortion is accompanied by a smaller ~4 Å shift of the acceptor arm of the P-site tRNA away from the PTC (Fig. 5d), whereas for other ARE–ABCF complexes, the CCA-end of the P-site tRNA completely vacates the PTC due to the presence of the longer ARD (as illustrated here for LsaA in Fig. 5c). Although PoxtA contains a shorter ARD than other ARE–ABCFs, the loop of the ARD still contacts the acceptor stem of the P-tRNA, precluding canonical interactions between the tRNA and the ribosome (Fig. 5d). As a consequence, the single-stranded CCA-3ʹ end of the P-site tRNA becomes contorted in the presence of PoxtA and the canonical interactions of the C75 and C74 of a P-site tRNA with 23S nucleotides G2251 and G2252, respectively, of the P-loop (H80) are disrupted (Fig. 5e, f). This results in a shift in register such that C75 basepairs with G2252 and C74 stacks upon Gln282 of PoxtA and interacts with G2253 and C2254 (Fig. 5f). The register shift is reminiscent of, but distinct to, that observed for the P_int_-tRNA in *E. coli* 70S ribosome complexes formed in the presence of a derivative of the antimicrobial peptide apidaecin and the termination release factor RF3^38^.

A comparison of the binding position of the ARD of PoxtA with that of the phenicol (chloramphenicol) and oxazolidinone (linezolid) antibiotics reveals that there is no steric overlap between PoxtA and either drug-binding site (Fig. 5b). Indeed, His285 of PoxtA, which comes closest to the drugs, is still located >16 Å away, and furthermore the ARD of PoxtA is partitioned from the drugs by the CCA-3ʹ end of the P-site tRNA (Fig. 5b). This raises the question as to whether PoxtA dislodges these drugs from the ribosome using an indirect mechanism, such as by inducing conformational changes within the 23S rRNA nucleotides comprising the drug-binding site(s), as proposed for other ARE–ABCFs^13–15^. To examine this, we compared the conformation of the PTC nucleotides comprising the drug-binding sites in our structures with those in the presence of chloramphenicol and linezolid. We could find no evidence that the presence of PoxtA induces any conformational changes within the drug-binding sites that would lead to dissociation of the drugs (Supplementary Figs. 12 and 13). In fact, we note that the conformation of the A-site pocket where the drugs bind is very similar, if not identical, for states I–IV, regardless of whether PoxtA is present (state I–III) or absent (state IV), or whether the A-site is vacant (state I–II, IV) or occupied (state III) (Supplementary Figs. 12 and 13). Although this suggests that PoxtA does not dislodge the drugs from the ribosome by altering the 23S rRNA in the binding site, we cannot exclude that such conformational changes occur upon ATP hydrolysis or upon dissociation of PoxtA from the ribosome.

An alternative scenario is that the distortion of the P-site tRNA induced by PoxtA binding indirectly reduces the affinity of the drugs for their A-site binding pocket. Both chloramphenicol and linezolid are context-specific inhibitors of translation elongation, such that the highest translation arrest activity occurs when the penultimate amino acid (−1 position) attached to the CCA-end of the P-site tRNA is alanine, and to a lesser extent, serine or threonine^27–29^. Structures of chloramphenicol on the ribosome with peptidyl-tRNA mimics reveal an intimate interaction between the drug and the nascent polypeptide chain, illustrating how alanine in the −1 position stabilises drug binding via a CH-π interaction (Fig. 5g)^39^. Since the distortion of the P-site tRNA by PoxtA involves a shift out of the PTC by 4 Å, effectively altering the nascent chain register by one amino acid, this would also result in a shift of the alanine away from chloramphenicol (Fig. 5h) and thereby perturb the interactions and reduce the affinity of the drug for the ribosome.

## Discussion

**Fig. 6:**
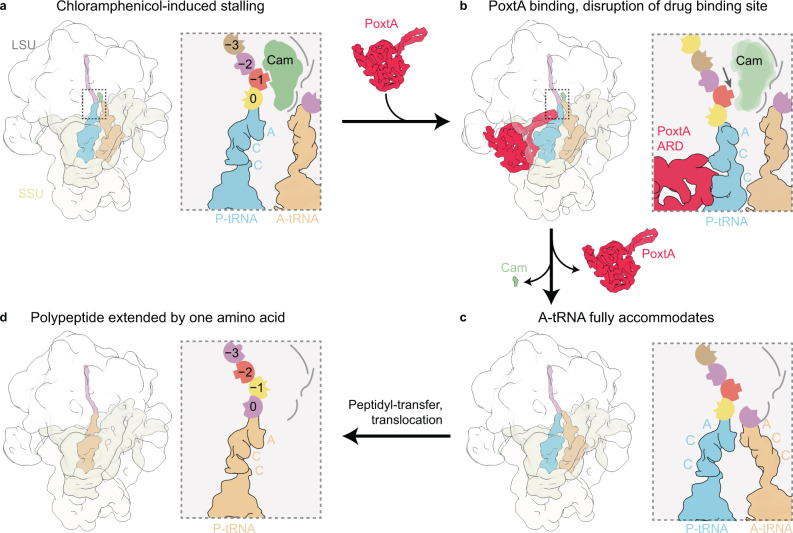
Model for ribosome protection by PoxtA. **a** Elongating ribosomes are stalled by PhO antibiotics with a peptidyl-tRNA in the P-site. The sidechain of the amino acid at position −1 contributes to the PhO-binding site. **b** Stalled ribosomes are recognised by PoxtA, which induces a shifted P-site tRNA and nascent chain, thereby disrupting the PhO-binding site. **c** After dissociation of the PhO drug and PoxtA, the P-tRNA and nascent chain return to the regular conformation. Accommodation of an A-tRNA occludes the drug-binding site. **d** After peptidyl transfer and translocation, amino acid at position −1 would change, thereby resetting the PhO-binding site.

Based on the structures of the PoxtA–ribosome complexes determined here, as well as the available literature on the mechanism of action of oxazolidinones and phenicols, we propose a model for how PoxtA can confer antibiotic resistance to these antibiotic classes (Fig. 6). As mentioned, linezolid and chloramphenicol are context-specific inhibitors that stall the ribosome during translation elongation with the peptidyl-tRNA in the P site and the drug bound within the A-site pocket of the PTC (Fig. 6a)^27–29^. The amino acid in the −1 position of the nascent polypeptide chain influences the strength of the arrest. Specifically, alanine elicited the strongest arrest^27–29^, apparently due to direct interaction with the ribosome-bound drug, as was recently shown for both chloramphenicol (Fig. 6a)^39^ and the oxazolidinones linezolid and radezolid^40^.

We propose that PoxtA recognises these drug-arrested ribosomes and binds to the vacant E-site (Fig. 6b). In contrast to other ARE–ABCFs where the CCA-end is completely removed from the PTC^13–15^, the binding of PoxtA causes the CCA-end to shift by only ~4 Å. Although modest, such a shift would be sufficient to change the register of the nascent polypeptide chain with respect to the drug, such that the drug can no longer interact with the amino acid in the −1 position, thereby decreasing its affinity for the ribosome and leading to its dissociation (Fig. 6b). It is possible that the shift of the nascent chain upon distortion of the P-site tRNA by PoxtA also contributes to “brushing” the drug from the ribosome^7^, analogous to the mechanism proposed for leader peptides that confer resistance to macrolides^41^. We also cannot exclude the possibility that conformational changes in PoxtA upon ATP hydrolysis play a role in drug release, in addition to facilitating dissociation of PoxtA from the ribosome (Fig. 6c).

What prevents PhO antibiotics from rebinding following their dissociation by PoxtA? As suggested previously for other ARE–ABCFs^13,15^, we favour a model where, following PoxtA dissociation, the peptidyl-tRNA can reaccommodate in the P site, thus allowing accommodation of a tRNA in the A site, which, in turn, would occlude the PhO-binding site (Fig. 6c). In this respect, we note that in contrast to previous ARE–ABCF structures^13,15^, we observe here a subpopulation of PoxtA–70S complexes where the A-site tRNA is fully accommodated at the PTC despite the presence of PoxtA and the distorted P-site tRNA. Thus, once the drug has been released, the A-site tRNA could accommodate at the PTC and following peptide bond formation and translocation, the context of the nascent chain would be shifted by one amino acid i.e. alanine in the −1 position would now be located at the −2 position and therefore disfavour drug rebinding (Fig. 6d). PoxtA and OptrA are often found on the same mobile genetic element as drug efflux pumps^22^, which may also contribute to preventing drug rebinding by transporting the dissociated drug directly out of the cell.

Our insights into the mechanism of action of PoxtA also provide a possible explanation for why these ARE–ABCFs do not confer resistance to MS_B_ or PLS_A_ antibiotics. Firstly, the ARD of PoxtA is too short to sterically overlap with these drugs and directly displace them from the ribosome, and secondly, binding of PoxtA does not perturb the rRNA portion of the drug-binding sites and therefore could not induce their dissociation by conformational relays in the 23S rRNA. For PLS_A_ antibiotics, which stall translation after initiation, there is no nascent chain that forms part of the drug-binding site. In the case of MS_B_ antibiotics, which stall translation elongation but bind deeper in the nascent peptide exit channel, perhaps the “pulling” effect of PoxtA on the nascent chain is mitigated by conformation elasticity of the several amino acids between the P-tRNA and the drug-binding site. Interestingly, ARE–ABCF antibiotics, such as MsrE or LsaA that confer resistance to MS_B_ and PLS_A_ antibiotics, respectively, do not confer resistance to PhO antibiotics, despite direct overlap between the ARDs and the drug-binding sites. We speculate that in these cases, the peptidyl-tRNA of the PhO-stalled ribosomes are refractory to the action of these ARE–ABCFs to distort the P-site tRNA; however, further investigations will be needed to validate this.

Given the similarity in ARD length and antibiotic spectrum, we believe that the findings and model presented here for PoxtA are likely to also be applicable for OptrA. Indeed, like OptrA, our MIC analysis provides no evidence for PoxtA conferring resistance to tetracycline antibiotics, consistent with the binding site of PoxtA in the E-site being far from the tetracycline-binding site located in the decoding A-site of the small subunit. We also see no evidence for conformational differences in the 16S rRNA nucleotides that comprise the tetracycline-binding site in the absence or presence of PoxtA. For this reason, we suggest reassigning letters from the PoxtA acronym from phenicol-oxazolidinone tetracycline A to phenicol-oxazolidinone transmissible A, analogous to OptrA.

## Methods

### Identification of poxtA EF9F6 and characterisation of *E. faecium* 9-F-6 antibiotic susceptibility

The *poxtA* EF9F6 gene was identified in the multidrug-resistant ST872 *E. faecium* 9-F-6 isolated in 2012 from faeces of a patient in a Norwegian hospital who had recently also been hospitalised in India^31^. ST872 is a single locus variant of ST80 which is a pandemic hospital-adapted genetic lineage. Transferable linezolid resistance (*optrA* and *cfr(D)* has recently been described in blood culture *E. faecium* ST872 strain from Australia^42^^.^ Species identification was performed by MALDI-TOF (Bruker, Billerica, USA) according to the manufacturer’s instructions and later confirmed by whole-genome sequencing^31^. Antimicrobial susceptibility testing was performed by broth microdilution using the EUENCF Sensititre plate (Thermo Fisher Scientific, Waltham, Massachusetts, USA), and further by gradient tests for vancomycin, teicoplanin, ampicillin, clindamycin, chloramphenicol and gentamicin (MIC Test strip, Liofilchem, Roseto Degli Abruzzi, Italy). The results (MICs) were interpreted according to EUCAST clinical breakpoints v. 10.0 2020 and MICs were within the accepted range for quality control strain *E. faecalis* ATCC 29212. The *E. faecium* 9-F-6 is resistant to linezolid (MIC = 8 mg/L), ampicillin (>256 mg/L), ciprofloxacin (>16 mg/L), high-level gentamicin (HLGR > 256 mg/L), high-level streptomycin (HLSR > 1024 mg/L) and high-level glycopeptides (vancomycin >256 mg/L and teicoplanin 128 mg/L), but susceptible to quinupristin/dalfopristin (MIC 4 mg/L) and tigecycline (MIC 0.12 mg/L) (Supplementary Table 1). Whole-genome sequences confirmed *vanA* and the *aac(6’)*-*Ie-aph(2”)-Ia* genes conferring high-level glycopeptide and gentamicin resistance, respectively, as well as a defunct, functionally inactive *cfr* pseudogene.

### Strains and plasmids

All bacterial strains and plasmids used in the study are listed in Supplementary Table 3. *E. faecalis* TX5332 (Rif^r^ Fus^r^ Kan^r^; *lsa* gene disruption mutant (OG1RF *lsa*::pTEX4577)) is a *lsa* knockout strain that was received from Barbara Murray^32^. pCIE_spec_ plasmid was constructed on the basis of the pCIE_cam_ plasmid, where the chloramphenicol resistance gene was substituted with spectinomycin, due to intrinsic resistance of PoxtA to phenicol antibiotics. All cloning was performed by the PEP facility (Umeå University). Plasmids encoding OptrA-ST16 (ref. ^21^) and OptrA-E35048 (ref. ^30^) were kindly provided by Anette M. Hammerum and Alberto Antonelli, respectively. In each case, the *optrA* gene was amplified with ribosome-binding sequence (RBS) (TAAGAGGAGGAGATAAAC) and inserted into pCIE_spec_ plasmid, resulting in pCIE_spec_:*optrA-ST16* and pCIE_spec_:*optrA-E35048*, respectively. The *poxtA AOUC-0915* gene^25^ was PCR amplified from genomic DNA of *S. aureus* AOUC-0915 (kindly provided by Alberto Antonelli) and inserted in pCIE_spec_ plasmid using the *Bam*HI and *Hin*dIII restriction sites. The RBS (TAAGAGGAGGAGATAAAC) was inserted directly in front of the ATG to improve expression levels. Genetic material encoding PoxtA-EF9F6 (OZN12776.1) was obtained from an *E. faecium* (9-F-6) isolated from a Norwegian patient in 2012, the genome of which has been sequenced (GCA_002263195.1)^31^. The *poxtA-EF9F6* gene with added RBS was inserted in the pCIE_spec_ plasmid resulting in pCIE_spec_:*poxtA-EF9F6*. These plasmids were used for MIC determinations. For use in pull-down experiments we introduced His_6_-TEV-FLAG_3_ tags at the C-terminus of either OptrA or PoxtA protein, with and without EQ mutations (E184Q and E471Q for PoxtA EQ_2_, E190Q and E470Q for OptrA EQ_2_, E470Q for OptrA EQ_1_) expressed from either pCIE_cam_ (OptrA variants) or pCIE_spec_ (PoxtA variants) plasmid.

### Bacterial transformation

*E. faecalis* electrocompetent cells were prepared as described previously^43^. Briefly, an overnight culture *E. faecalis* TX5332 grown in the presence of 2 mg/mL of kanamycin was diluted to OD_600_ of 0.05 in 50 mL of Brain Heart Infusion (BHI) media and grown further to an OD_600_ of 0.6–0.7 at 37 °C with moderate shaking (160 rpm/10 × *g*). Cells were collected by centrifugation at 4000 rpm (1200 × *g*) at 4 °C for 10 min. Cells were resuspended in 0.5 mL of sterile lysozyme buffer (10 mM Tris-HCl pH 8; 50 mM NaCl, 10 mM EDTA, 35 µg/mL lysozyme), transferred to 0.5 mL Eppendorf tube and incubated at 37 °C for 30 min. Cells were pelleted at 10,000 rpm (8000 × *g*) at 4 °C for 10 min and washed three times with 1.5 mL of ice-cold electroporation buffer (0.5 M sucrose, 10% (w/v) glycerol). After the last wash, the cells were resuspended in 500 µL of ice-cold electroporation buffer and aliquoted and stored at −80 °C. For electroporation, 35 µL of electrocompetent cells were supplemented with 0.5–1 µg of plasmid DNA, transferred to ice-cold 1 mm electroporation cuvette and electroporated at 1.8 keV. Immediately after electroporation 1 mL of ice-cold BHI media was added to the cells, the content of the cuvette was transferred to 1.5-mL Eppendorf tubes and the cells were recovered at 37 °C for 2.5 h and plated to BHI plates containing appropriate antibiotics.

### Antibiotic susceptibility testing

*E. faecalis* cells were grown in BHI media supplemented with 2 mg/mL kanamycin (to prevent *lsa* revertants), 0.1 mg/mL spectinomycin (to maintain the pCIE_spec_ plasmid), 100 ng/mL of cCF10 peptide (to induce expression of proteins of interest) as well as increasing concentrations of antibiotics. The media was inoculated with 5 × 10^5^ CFU/mL (OD_600_ of approximately 0.0005) of *E. faecalis* Δ*lsaA* (*lsa::Kan*) strain TX5332 transformed either with empty pCIE_spec_ plasmid or with pCIE_spec_ encoding indicated protein of interest. After 16–20 h at 37 °C without shaking, the presence or absence of bacterial growth was scored by eye.

### Preparation of bacterial biomass

*For cryo-EM: E. faecalis* TX5332 transformed with pCIE_spec_/pCIE_cam_-based expression constructs (either empty vector or expressing wild-type/EQ_2_ variants of both PoxtA-HTF and OptrA-HTF as well as EQ_1_ variant of OptrA-HTF) were grown overnight from a single colony in BHI media supplemented with appropriate antibiotics (100 µg/mL of spectinomycin for pCIE_spec_-based constructs, 10 µg/mL chloramphenicol for pCIE_cam_-based constructs). Overnight cultures were then diluted to a starting OD_600_ of 0.05 in 200 mL of the same media. Cells were grown with intensive shaking at 37 °C till OD_600_ of 0.6 and were induced with 100 ng/mL of cCF10 peptide for 30 min prior harvesting by centrifugation at 10,000 × *g* for 15 min at 4 °C.

*For 5Pseq: E. faecalis* TX5332 Δ*lsa*::*kan* was transformed with either the empty vector pCIE_spec_ (VHp426) or a vector expressing *poxtA*-EF9F6 (VHp506). 50 mL cultures were inoculated from an overnight culture to 0.005 OD_600_, in filtered BHI media containing 100 ng/mL cCF10 peptide (to induce *poxtA* expression) and the cells were grown at 37 °C with shaking at 160 rpm (10 × *g*). Once cells reached 0.3 OD_600_ the cells were treated with either linezolid at 4 µg/mL (final concentration) or corresponding volume of ethanol (for as the untreated control) for 10 min, harvested via centrifugation (4000 rpm/1200 × *g*, 2 min), and flash-frozen in liquid nitrogen for storage at −80 °C and subsequent processing.

### RNA extraction

RNA was extracted via phenol-chloroform extraction^44^. Briefly, 300 μL LET-phenol (LET: 25 mM Tris-HCl pH 8.0, 100 mM LiCl, 20 mM EDTA) was added to cell pellets and vortexed with an equal volume of 0.1-mm glass beads to lyse cells. In total, 250 μL of water and 250 μL of acidic phenol/chloroform was added before vortexing for a further 2 min. RNA was then extracted from the aqueous phase with an acidic phenol/chloroform step followed by a final chloroform extraction step. RNA was then ethanol precipitated in the presence of NaOAc (pH 5.2). RNA was assessed by running a 2% agarose gel and quantified by fluorescence (Qubit, Thermo Fisher Scientific).

### 5Pseq library preparation and data analysis

HT-5Pseq libraries were prepared as described earlier^45^. Briefly, 4 µg total RNA from *E. faecalis* was DNase treated (TURBO Dnase kit, Thermo Fisher Scientific), ligated overnight at 16 °C to r5P_RNA_ oligo (CrArCrGrArCrGrCrUrCrUrUrCrCrGrArUrCrUrNrNrNrNrNrNrNrN) and subsequently purified using 1.8 volumes of RNAClean XP beads (Beckman Coulter). Ligated RNA was then reverse transcribed using random hexamer (5Pseq-RT, GTGACTGGAGTTCAGACGTGTGCTCTTCCGATCTNNNNNN, 20 µM) to prime. Leftover RNA was degraded using NaOH. Ribosomal RNA was removed using previously described rRNA DNA oligo depletion mixes^33^, following duplex-specific nuclease (DSN, Evrogen) degradation. rRNA-depleted cDNA was amplified by PCR (17 cycles) using Illumina dual index (i5, i7) PCR primers (Supplementary Table 4) and enriched for fragments with the range of 300–500 nt using Ampure XP. Size-selected HT-5P Libraries were quantified by fluorescence (Qubit, Thermo Fisher Scientific), size estimated using an Agilent Bioanalyzer and sequenced using a NextSeq2000 Illumina sequencer (100 cycles P3 kit). BCL sequencing files were demultiplexed and converted to fastq using bcl2fastq software (bcl2fastq v2.20.0.422) with default settings (allowing for 1 barcode-mismatch, minimum trimmed read length: 35, mask short adapter reads: 22). FASTQC and MultiQC (v1.9) tools were used for quality control. In addition to standard Illumina dual index (i5, i7), UMI barcode was extracted using UMI-tools (version 1) with default options (using –bc-pattern NNNNNNNN)^46^, and reads were mapped to *E. faecalis* genome index (build GCF003319925.1_ ASM331992v) using STAR (version 2.7.2b, options –alignEndsType Extend5pOfRead1; --outFilterMatchNminOverLread 0.9; --outFilterMultimapNmax 3; --limitBAMsortRAM 100000000000; alignIntronMax 2500)^47^. Aligned reads were deduplicated (i.e. potential artefactual PCR duplicates are removed of the analysis) with UMI-tools (version 1, options --soft-clip-threshold 1 --edit-distance 2 --method unique). Alignment BAM files were supplied as input to the *fivepseq* application for 5P-end distribution analysis and figure generation^48^. To generate the heat maps, cumulative counts at relative distance from each amino acid were taken and normalised to library size to derive RPM values, then the log_2_-fold change of the RPM values between the experimental condition (linezolid treatment or PoxtA expression) and the untreated control was used to compare the pairs of samples in each case.

### Preparation of clarified lysates

Cell pellets were resuspended in 1.5 mL of cell opening buffer (95 mM KCl, 5 mM NH_4_Cl, 20 mM HEPES pH 7.5, 1 mM DTT, 5 mM Mg(OAc)_2_, 0.5 mM CaCl_2_, 8 mM putrescine, 0.5–0.75 mM ATP, 1 mM spermidine, 1 tablet of cOmplete™ EDTA-free Protease Inhibitor Cocktail (Roche) per 10 mL of buffer). Resuspended cells were opened by FastPrep homogeniser (MP Biomedicals) with 0.1-mm Zirconium beads (Techtum) in four cycles by 20 s with a 1 min chill on ice. Cell debris was removed after centrifugation at 14,800 × *g* for 15 min at 4 °C. Total protein concentration in the supernatant was measured by Bradford assay (Bio-Rad), the supernatant was aliquoted and frozen in liquid nitrogen.

### Affinity purification on anti-FLAG M2 affinity gel

In all, 100 µL of well-mixed anti-FLAG M2 Affinity Gel aliquots (Sigma) were loaded on columns (Micro Bio-Spin Columns, Bio-Rad) and washed twice with 1 mL of cell opening buffer by gravity flow. All incubations, washings and elutions were done at 4 °C. The total protein concentration of each individual lysate was separately adjusted to 2 mg/mL with cell opening buffer pH-adjusted with either HEPES pH 7.5 (OptrA samples) or glycine pH 9.0 (PoxtA samples). The pH of the opening buffer was selected through a series of small-scale pull-down experiments aimed at optimisation of the pull-down efficiency. 1 mL of each lysate was loaded on columns and incubated for 2 h with end-over-end mixing for binding. The columns were washed five times with 1 mL of cell opening buffer (pH 7.5 in the case of OptrA samples, pH 9.0 in the case of PoxtA samples) by gravity flow. For elution of FLAG-tagged proteins and their complexes 100 µL of 0.2 mg/mL FLAG_3_ peptide (Sigma) was added to samples, the solutions were incubated at 4 °C for 20 min with end-over-end mixing. Elutions were collected by centrifugation at 2000 × *g* for 2 min at 4 °C. 20 µL aliquots of collected samples (flow-through, washes and elutions) were mixed with 5 µL of 5× SDS loading buffer and heated up at 95 °C for 15 min. The beads remaining in the column were resuspended in 100 µL of 1× SDS loading buffer. Denatured samples were loaded on 15% SDS-PAGE. SDS-gels were stained by “Blue-Silver” Coomassie Staining^49^ and destained in water for at least one hour (or overnight) before imaging with LAS4000 (GE Healthcare).

### Preparation of cryo-EM grids

Elutions from pull-downs were kept on ice until being applied within 2 h to glow discharged cryo-grids (Quantifoil 2/2 Cu_300_ coated with 2 nm continuous carbon). In total, 3.5 µL of sample was loaded on grids three times manually at room temperature conditions and a fourth time in Vitrobot (FEI) under conditions of 100% humidity at 4 °C, blotted for 5 s and vitrified by plunge-freezing in liquid ethane. Samples were imaged on a Titan Krios (FEI) operated at 300 kV at a nominal magnification of ×165,000 (0.86 Å/pixel) with a Gatan K2 Summit camera at an exposure rate of 5.85 electrons/pixel/s with a 4 s of exposure and 20 frames using the EPU software.

### Single-particle reconstruction

Processing was performed in RELION 3.1 unless otherwise specified^50^. MotionCor2 with 5 × 5 patches and CTFFIND4 (using power spectra) were used for motion correction and initial CTF estimation^51,52^. Micrographs with estimated CTF fits beyond 4.5 Å and CTF figure of merits >0.04 were selected for further processing. Particles were picked with crYOLO using the general model^53^. After 2D classification, all ribosome-like classes were selected, particles extracted with a 3× reduced pixel size (2.46 Å), and an initial model created ab initio. After 3D refinement using the ab initio model as a reference^54^, 3D classification with eight classes and without angular searching was performed. The majority of particles (~80%) clustered into two classes that contained protein-like density in the E-site, and which were selected for further processing. Particles were re-extracted at the original pixel size, and serial 3D refinements with CTF refinement and Bayesian polishing^55^ were performed until the resolution did not improve further, resulting in the “combined 70 S volume”. A mask around the A, P and E sites was created and used for partial signal subtraction with re-extraction at a pixel size of 2.46 Å. These particles were used for 3D classification with six classes, *T* = 40 and the resolution of the expectation step limited to 12 Å. Four of the six resulting classes, labelled states I–IV, were chosen for refinement with the original pixel size. For the multibody refinement of the combined 70S class^56^, volumes corresponding to the LSU core, CP, SSU body, SSU head and E-site were isolated using the volume eraser tool in UCSF ChimeraX^57^, and masks created from the densities low-pass-filtered to 30 Å. Bsoft was used to estimate local resolution^58^.

### Molecular modelling

Molecular models were created/adjusted with Coot^59^ and ISOLDE^60^, and refined with Phenix^61^ against unsharpened maps. A previous structure of the *E. faecalis* 70S ribosome in complex with the ABCF-ARE LsaA (PDB 7NHK)^15^ was used as the starting model for the ribosome, initially into multibody-refined maps from the combined 70S volume. PDB IDs 7K00 (ref. ^62^) and 6O90 (ref. ^37^) were also used as templates in parts of the 70S, and PDB ID 3U4M^63^ was used as a template for the L1 stalk region. Likely metal ions were assigned by the presence of typical coordination shapes, as well as strength of density and estimated coordination distances. However, we caution that these assignments could not always be made unambiguously. Phenix.douse was used to place water molecules. A recent structure of the *S. aureus* ribosome with modified nucleotides (PDB ID 6YEF)^64^ was compared to the density to check for RNA modifications, and where the density matched the modification with high confidence, that modification was inserted into the *E. faecalis* 70S model. We note that, despite the use of an inactivating resistance marker, density for the selection agent spectinomycin was observed in each map. Spectinomycin was modelled based on PDB ID 4V56 (ref. ^65^). For PoxtA, homology models were generated by SWISS-MODEL^66^ using the crystal structure of EttA (PDB ID 4FIN)^12^ and the previous cryo-EM structure of LsaA (PDB ID 7NHK)^15^. The ARD was sufficiently well-resolved that it could be built manually. The Arm insertion in NBD1 was built manually with low confidence (especially in the loop connecting the two α-helices). PSI-PRED secondary structure predictions were used to help define secondary structure boundaries^67^. The models for states I–IV were assembled using the model from the combined 70S as a template. For the A-tRNA in state III, tRNA-Lys from PDB ID 5E7K (ref. ^68^) was used as a template for *E. faecalis* tRNA-Lys-UUU-1-1 (gtRNAdb nomenclature^69^) based on density.

### Sequence analysis methods

Representative ABCF sequences were aligned with Mafft L-INS-i 7.453 (ref. ^70^) and visualised with Jalview 2.11.1.4 (ref. ^71^) and Aliview 1.26 (ref. ^72^). Phylogenetic analysis was carried out with IQTree version 2.1.2 on the CIPRES server with 1000 rapid bootstrap replicates and automatic model detection^73,74^. Positions with more than 50% gaps were removed with TrimAL v1.4.rev6 before phylogenetic analysis^75^.

### Figures

Figures were created using UCSF ChimeraX, PyMol v2.4, and assembled with Adobe Illustrator (Adobe Inc.) and Inkscape (latest development release, regularly updated). For difference vectors, the PyMol modevectors.py script (modified by Jamie Cate, University of California, Berkeley, USA) was used. For measuring SSU rotation compared to the LsaA–70S volume, the SSU head and body from PoxtA combined 70S volume were separately aligned to the LsaA–70S model, then used to draw the vectors.

### Reporting summary

Further information on research design is available in the Nature Research Reporting Summary linked to this article.

## Supplementary information

Supplementary Information

Reporting Summary

## Data Availability

Micrographs have been deposited as uncorrected frames in the Electron Microscopy Public Image Archive (EMPIAR) with the accession codes EMPIAR-10764. Cryo-EM maps have been deposited in the Electron Microscopy Data Bank (EMDB) with accession codes EMD-13241 (*E. faecalis* combined 70S volume), EMD-13242 (PoxtA–70S state I), EMD-13243 (PoxtA–70S state II), EMD-13244 (PoxtA–70S state III with A-site tRNA) and EMD-13245 (*E. faecalis* 70S with P-tRNA state IV). Molecular models have been deposited in the Protein Data Bank with accession codes 7P7Q (*E. faecalis* combined 70S volume), 7P7R (PoxtA–70S state I) 7P7S (PoxtA–70S state II), 7P7T (PoxtA–70S state III with A-site tRNA) and 7P7U (*E. faecalis* 70S with P-tRNA, state IV). Source data are provided with this paper. Sequencing data are deposited at GEO with accession code GSE179348. Source data are provided with this paper.

## Source data

Source Data
