# Supplementary material for: Structural basis for PoxtA-mediated resistance to phenicol and oxazolidinone antibiotics
Source: Nat Commun. 2022 Apr 6;13:1860. doi: 10.1038/s41467-022-29274-9 (PMC8987054; doi:10.1038/s41467-022-29274-9)
Supplement: Supplementary file 1 — Supplementary Information [file 41467_2022_29274_MOESM1_ESM.pdf]

SUPPLEMENTARY INFORMATION FOR

Structural basis for PoxtA-mediated resistance to Phenicol and Oxazolidinone antibiotics

Caillan Crowe-McAuliffe[1,#], Victoriia Murina[2,3,4,#], Kathryn Jane Turnbull[2,5], Susanne Huch[6], Marje Kasari[2,3,7], Hiraku Takada[2,3,8], Lilit Nersisyan[6], Arnfinn Sundsfjord[9,10], Kristin Hegstad[9,10], Gemma C. Atkinson[3,11], Vicent Pelechano[6], Daniel N. Wilson[1,*], Vasili Hauryliuk[2,3,4,7,11,*]

[1] Institute for Biochemistry and Molecular Biology, University of Hamburg, Martin-Luther-King-Platz 6, 20146 Hamburg, Germany.

[2] Laboratory for Molecular Infection Medicine Sweden (MIMS), Umeå University, 90187 Umeå, Sweden.

[3] Umeå Centre for Microbial Research (UCMR), Umeå University, 901 87 Umeå, Sweden

[4] Department of Molecular Biology, Umeå University, 90187 Umeå, Sweden.

[5] Department of Clinical Microbiology, Rigshospitalet, 2200, Copenhagen, Denmark.

[6] SciLifeLab, Department of Microbiology, Tumor and Cell Biology. Karolinska Institutet, 171 65 Solna, Sweden

[7] University of Tartu, Institute of Technology, 50411 Tartu, Estonia.

[8] Faculty of Life Sciences, Kyoto Sangyo University, Kamigamo, Motoyama, Kita-ku, Kyoto 603-8555, Japan.

[9] Department of Microbiology and Infection Control, Norwegian National Advisory Unit on Detection of Antimicrobial Resistance, University Hospital of North Norway, Tromsø, Norway.

[10] Research Group for Host-Microbe Interaction, Department of Medical Biology, Faculty of Health Sciences, UiT Arctic University of Norway, NO-9037 Tromsø, Norway.

[11] Department of Experimental Medical Science, Lund University, 221 00 Lund, Sweden.

[#] These authors contributed equally.

*Correspondence to:

Daniel Wilson (Daniel.Wilson@chemie.uni-hamburg.de) and Vasili Hauryliuk (vasili.hauryliuk@med.lu.se)

Supplementary Table 1. Broth microdilution (BMD) and gradient (GRAD) MIC testing of multidrug-resistant ST872 *E. faecium* 9-F-6.

	MIC, µg/mL		
	**EUCAST breakpoints**	**BMD**	**GRAD**
**Ampicillin**	S≤4 R> 8	**>32**	**>256**
**Amoxicillin**	S≤4 R> 8	**>32**	ND
**Imipenem**	S≤0,001 R> 4	**>16**	ND
**Ciprofloxacin**	S≤4 R> 4	**>16**	ND
**Levofloxacin**	S≤4 R> 4	**>16**	ND
**Gentamicin**	HLGR >128	**>256**	**>1024**
**Streptomycin**	HLSR > 512	**>1024**	ND
**Teicoplanin**	S≤2 R> 2	**>8**	**128**
**Vancomycin**	S≤4 R> 4	**>16**	**>256**
**Clindamycin**	N/A	ND	>256
**Quinupristin/Dalfopristin**	S≤1 R>4	4	ND
**Tigecycline**	S≤0.25 R>0.25	0.12	ND
**Linezolid**	S≤4 R> 4	**8**	**16**
**Chloramphenicol**	N/A	ND	16

MICs above the clinical break points, i.e. classifying the strain as resistant (R) are in bold, MICs below the break point, i.e. classifying the strain as susceptible (S) are in regular font. HLGR and HLSR stands for high-level gentamicin and streptomycin resistance. Note that BMD-based MICs overrule GRAD test MICs. ND stands for not determined, N/A stands for not applicable.

Supplementary Table 2. Cryo-EM data collection, modelling and refinement statistics.

	Combined 70S	State I	State II	State III	State IV
EMD ID	13241	13242	13243	13244	13245
PDB ID	7P7Q	7P7R	7P7S	7P7T	7P7U
**Data collection**					
Magnification (×)	165 000	165 000	165 000	165 000	165 000
Electron fluence ($e^{-}/Å^{2}$)	30.255	30.255	30.255	30.255	30.255
Defocus range (µm)	−0.5 to −1.5	−0.5 to −1.5	−0.5 to −1.5	−0.5 to −1.5	−0.5 to −1.5
Pixel size (Å)	0.82	0.82	0.82	0.82	0.82
Initial particles	203 321	203 321	203 321	203 321	203 321
Final particles	112 877	29 581	29 875	23 806	18 512
Average resolution (Å) (FSC threshold 0.143)	2.4	2.9	3.0	2.9	3.1
**Model composition**					
Atoms	151 218	147 589	147 589	149 096	139 841
Protein residues	6 096	6 097	6 097	6 097	5 337
RNA bases	4 625	4 625	4 625	4697	4 549
**Refinement**					
Map CC around atoms	0.91	0.91	0.91	0.91	0.90
Map CC whole unit cell	0.90	0.91	0.90	0.91	0.90
Map sharpening B factor ($Å^{2}$)	−46	−55	−59	−58	−59
R.M.S. deviations					
Bond lengths (Å)	0.005	0.005	0.005	0.004	0.004
Bond angles (°)	0.773	0.628	0.624	0.577	0.633
**Validation**					
MolProbity score	1.37	1.39	1.42	1.34	1.46
Clash score	4.76	4.52	5.59	4.57	6.17
Poor rotamers (%)	0.76	0.56	0.76	0.68	0.42
Ramachandran statistics					
Favoured (%)	97.35	97.05	97.40	97.43	97.37
Outlier (%)	0.02	0.03	0.02	0.03	0.08

Supplementary Table 3. Strains and plasmids used in this study.

Strain or Plasmid	VH lab database number	Description	Reference
E. faecium 9-F-6	N.A.	Multidrug-resistant clinical isolate	Ref.[31]
E. faecalis TX5332 Δ*lsaA* (*lsa*::Kan)	N.A.	Rif^r Fus^r Kan^r; *lsa* gene disruption mutant (OG1RF *lsa*::pTEX4577))	Ref.[32]
$pCIE_{spec}$	VHp426	pCIE, $Spec^r$; Cm^r gene swapped to spectinomycin resistance ($Spec^r$) gene	Ref.[15]
$pCIE_{spec}$:*poxtA-AOUC-0915*-HTF	VHp553	$pCIE_{spec}$ with optimized RBS expressing PoxtA AOUC-0915 with C-terminal His_6-TEV-$FLAG_3$ tag	This work
$pCIE_{spec}$:*poxtA-AOUC-0915*-EQ_2-HTF	VHp609	$pCIE_{spec}$ with optimized RBS expressing PoxtA AOUC-0915 with C-terminal His_6-TEV-$FLAG_3$ tag and $E_{184}Q$ and $E_{471}Q$ mutations	This work
$pCIE_{spec}$:*lsaA*	VHp431	$pCIE_{spec}$ with optimized RBS expressing LsaA	Ref.[15]
$pCIE_{spec}$:*optrA-ST16*	VHp432	$pCIE_{spec}$ with optimized RBS expressing OptrA ST16	This work
$pCIE_{spec}$:*optrA-E35048*	VHp506	$pCIE_{spec}$ with optimized RBS expressing OptrA-E35048	This work
$pCIE_{spec}$:*poxtA-EF9F6*	VHp504	$pCIE_{spec}$ with optimized RBS expressing PoxtA EF9F6	This work
$pCIE_{spec}$:*poxtA-AOU-0915*	VHp507	$pCIE_{spec}$ with optimized RBS expressing PoxtA AOUC-0915	This work
$pCIE_{Cam}$:*optrA-ST16-HTF*	VHp223	$pCIE_{Cam}$ with optimized RBS expressing OptrA ST16 with C-terminal His_6-TEV-$FLAG_3$ tag	This work
$pCIE_{Cam}$:*optrA-ST16-E470Q-HTF*	VHp294	$pCIE_{Cam}$ with optimized RBS expressing OptrA ST16 with C-terminal His_6-TEV-$FLAG_3$ tag and $E_{470}Q$ substitution	This work
$pCIE_{Cam}$:*optrA-ST16-EQ_2-HTF*	VHp295	$pCIE_{Cam}$ with optimized RBS expressing OptrA ST16 with C-terminal His_6-TEV-$FLAG_3$ tag and $E_{190}Q$ and $E_{470}Q$ substitutions	This work

Abbreviations used: spec – spectinomycin, RBS – ribosome binding site, cm – chloramphenicol, HTF – His_6-TEV-$FLAG_3$, ARD – antibiotic resistance domain. NA stands for not applicable.

Supplementary Table 4. Oligonucleotide primers used in for the 5PSeq library preparation.

Oligonucleotide	Sequence, 5'-3'
NEBi515	AATGATACGGCGACCACCGAGATCTACAC**TGTGGCAT**ACACTCTTTCCCTACACGACGCTCTTCCGATC*T
PE2_MPX_01	CAAGCAGAAGACGGCATACGAGAT**CGTGAT**GTGACTGGAGTTCAGACGTGTGCTCTTCCGATC*T
PE2_MPX_02	CAAGCAGAAGACGGCATACGAGAT**ACATCGG**TGACTGGAGTTCAGACGTGTGCTCTTCCGATC*T
PE2_MPX_03	CAAGCAGAAGACGGCATACGAGAT**GCCTAA**GTGACTGGAGTTCAGACGTGTGCTCTTCCGATC*T
PE2_MPX_04	CAAGCAGAAGACGGCATACGAGAT**TGGTCA**GTGACTGGAGTTCAGACGTGTGCTCTTCCGATC*T
PE2_MPX_09	CAAGCAGAAGACGGCATACGAGAT**CTGATC**GTGACTGGAGTTCAGACGTGTGCTCTTCCGATC*T
PE2_MPX_10	CAAGCAGAAGACGGCATACGAGAT**AAGCTA**GTGACTGGAGTTCAGACGTGTGCTCTTCCGATC*T
PE2_MPX_11	CAAGCAGAAGACGGCATACGAGAT**GTAGCC**GTGACTGGAGTTCAGACGTGTGCTCTTCCGATC*T
PE2_MPX_12	CAAGCAGAAGACGGCATACGAGAT**TACAAG**GTGACTGGAGTTCAGACGTGTGCTCTTCCGATC*T
PE2_MPX_13	CAAGCAGAAGACGGCATACGAGAT**TTGACT**GTGACTGGAGTTCAGACGTGTGCTCTTCCGATC*T
PE2_MPX_14	CAAGCAGAAGACGGCATACGAGAT**GGAACT**GTGACTGGAGTTCAGACGTGTGCTCTTCCGATC*T
PE2_MPX_15	CAAGCAGAAGACGGCATACGAGAT**TGACAT**GTGACTGGAGTTCAGACGTGTGCTCTTCCGATC*T
PE2_MPX_16	CAAGCAGAAGACGGCATACGAGAT**GGACGG**GTGACTGGAGTTCAGACGTGTGCTCTTCCGATC*T

IUPAC nucleotide codes are used to indicate oligonucleotides with degenerated bases. * indicates the S-linkage between the two bases. Barcodes are identified in bold.

Supplementary Figures

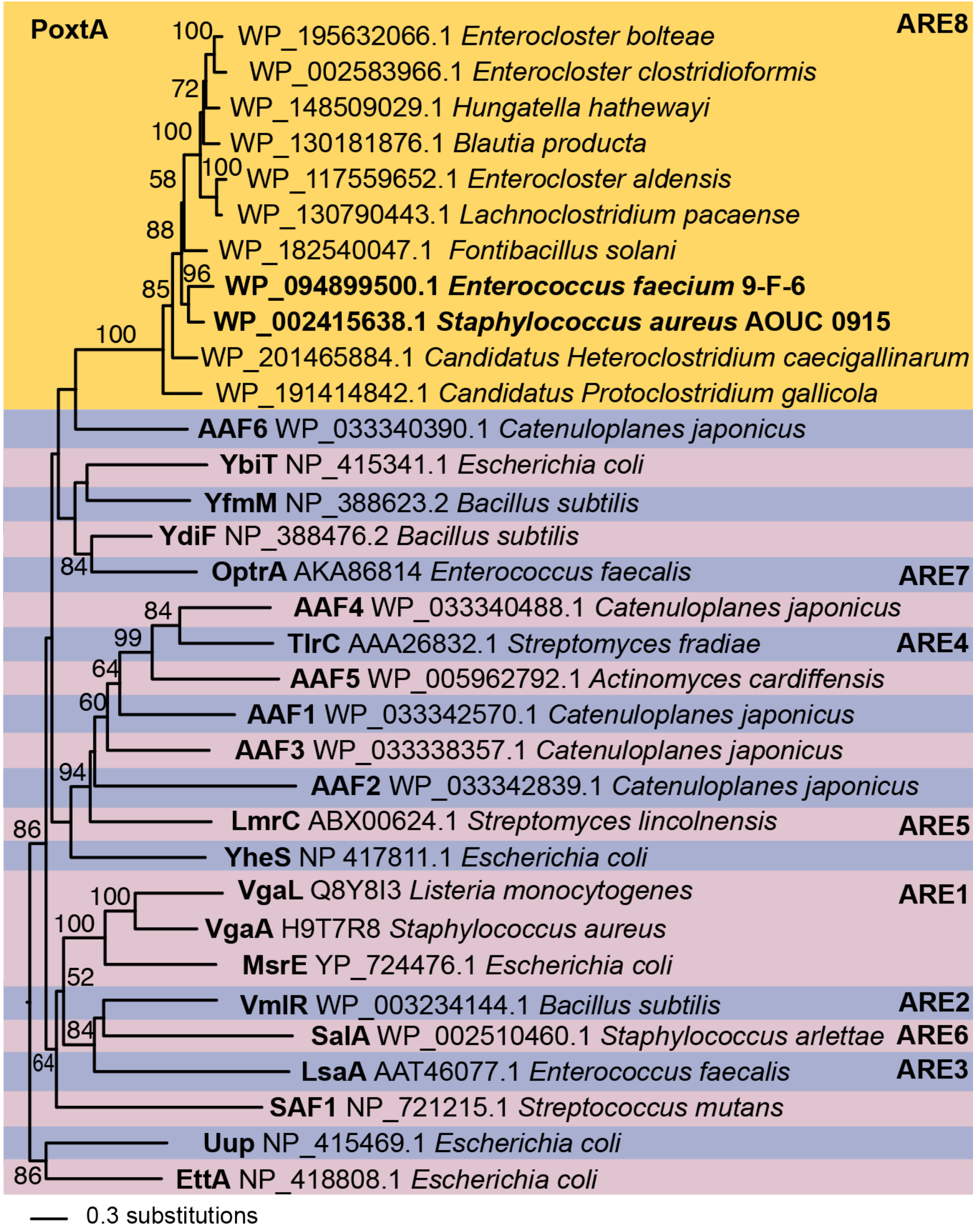

Supplementary Figure 1. ARE-ABCF phylogeny with a focus on PoxtA. The tree is a maximum likelihood phylogeny of selected ABCF representatives. Numbers on branches correspond to IQ-Tree ultra-fast bootstrap support in percentages[74], and branch length is proportional to the number of substitutions as per the scale bar in the lower left. The ARE8 subfamily comprising PoxtA-like proteins has 100% support, but its relationship with other subfamilies is unresolved. OptrA is most closely related to the YdiF subfamily of mostly vertically inherited and presumably housekeeping ABCF (84% bootstrap support). This suggests that the similar resistance spectrum of OptrA and PoxtA is a case of convergent evolution.

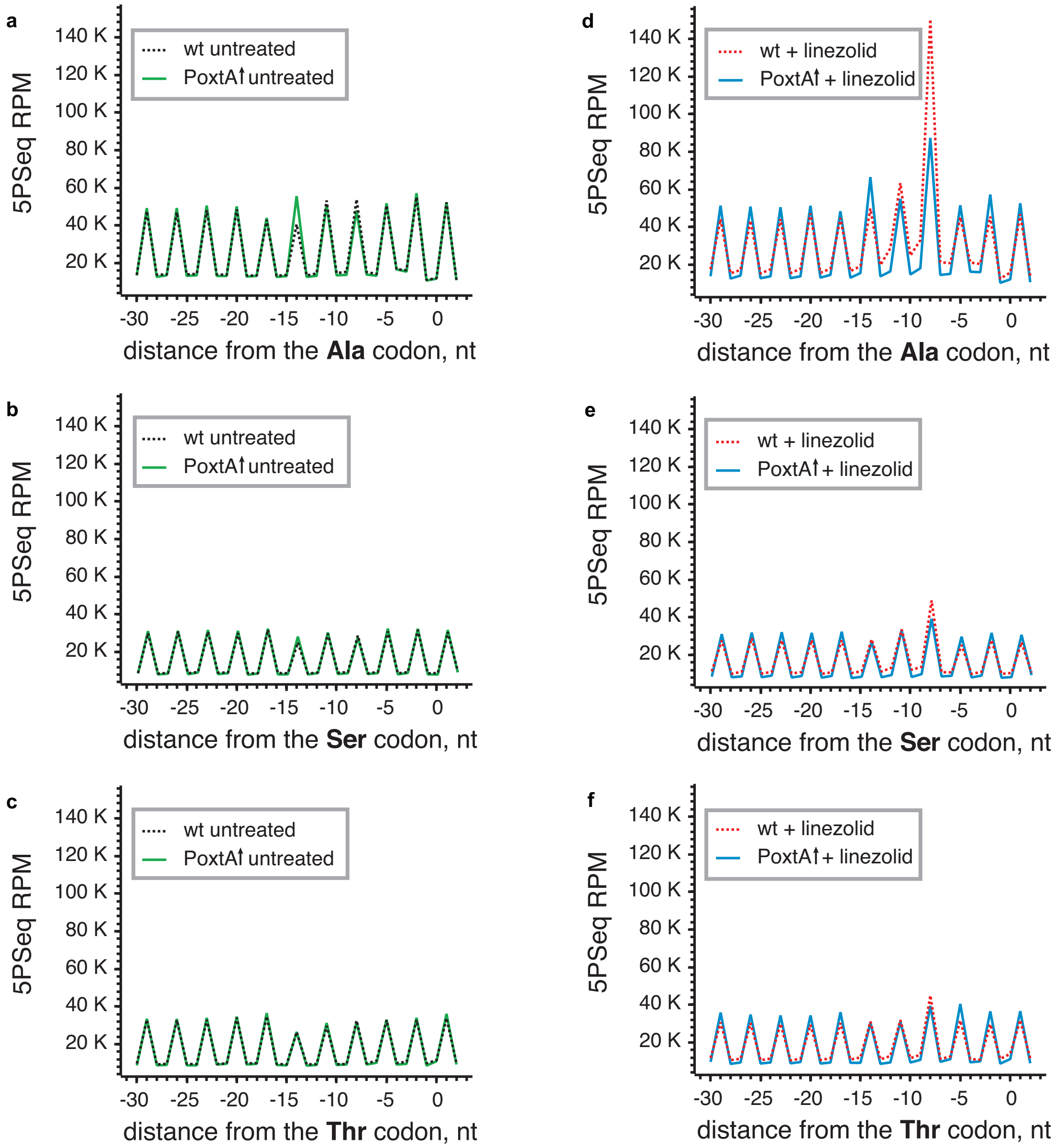

Supplementary Figure 2. PoxtA-mediated rescue of linezolid-induced ribosomal stalls. 5PSeq reveals ribosomal stalls upon treatment with linezolid with alanine, serine and threonine present at the –1 position on the nascent chain. **a**–**f** Metagene plots aligned to alanine (**a**, **d**) serine (**b**, **e**) or threonine (**c**, **f**) in from either *E. faecalis* harbouring the empty vector pCIE$_{spec}$ (**a**–**c**) or expressing PoxtA (indicated with up-pointing arrow) (**d**–**f**). Note, in all plots increased coverage at –8 nt is indicative of a ribosomal stall with the denoted amino acid present at position –1 of the nascent chain. All analysis was performed on pooled datasets from three replicates.

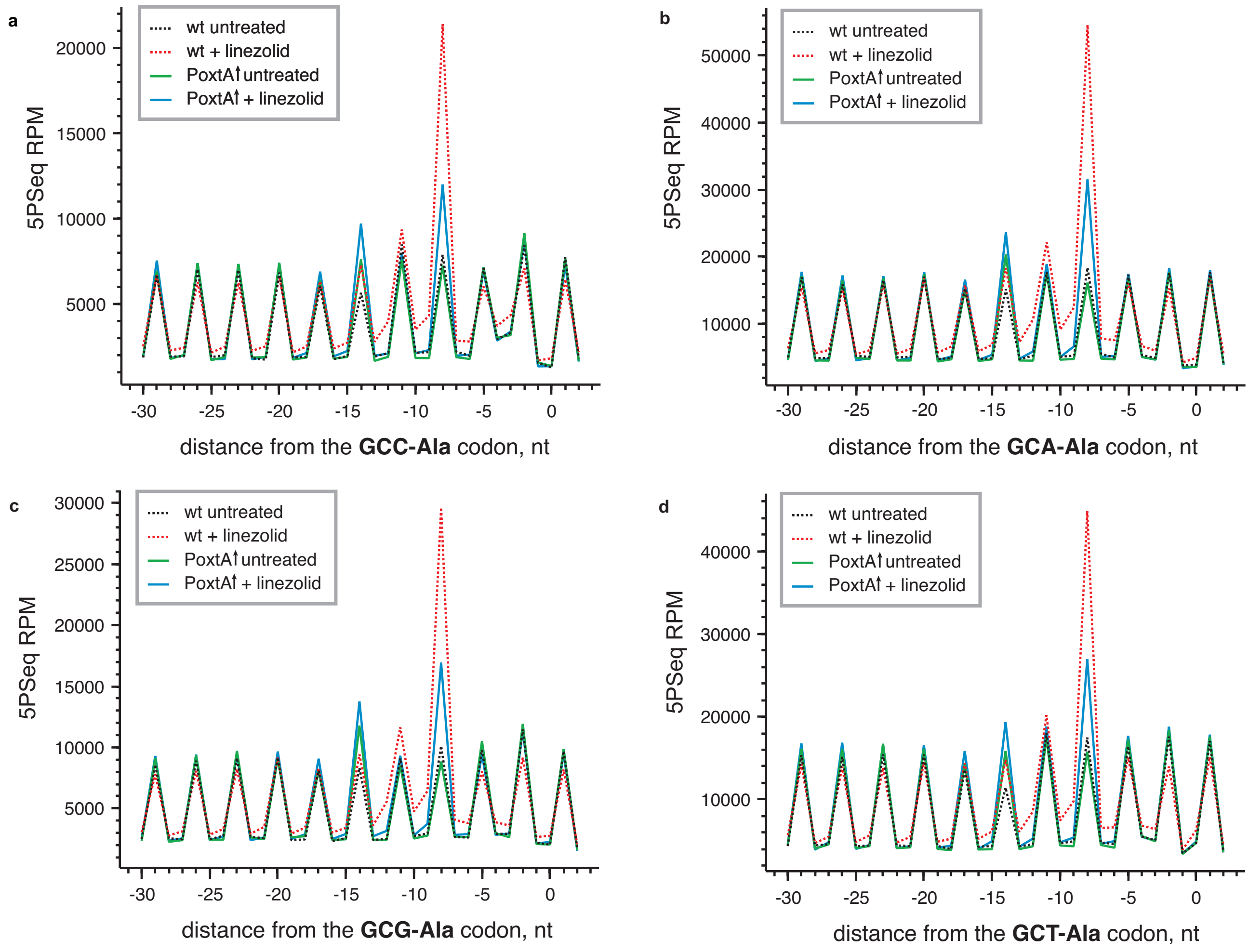

Supplementary Figure 3. Linezolid-induced ribosomal stalling is similarly pronounced on all alanine iso-codons. 5PSeq reveals ribosomal stalls upon treatment with linezolid with specific alanine-encoding codons GCC (**a**), GCA (**b**), GCG (**c**) and GCT (**d**), present in the E-site of the ribosome. Metagene plots from either wild-type *E. faecalis* harbouring the empty pCIE$_{spec}$ vector or expressing PoxtA (indicated with up-pointing arrow), with or without linezolid treatment. Note, in all plots, increased coverage at –8 nt is indicative of a ribosomal stall with the denoted codon in the ribosomal E-site and its decoded amino acid present at position –1 of the nascent chain. All analysis was performed on pooled datasets from three replicates.

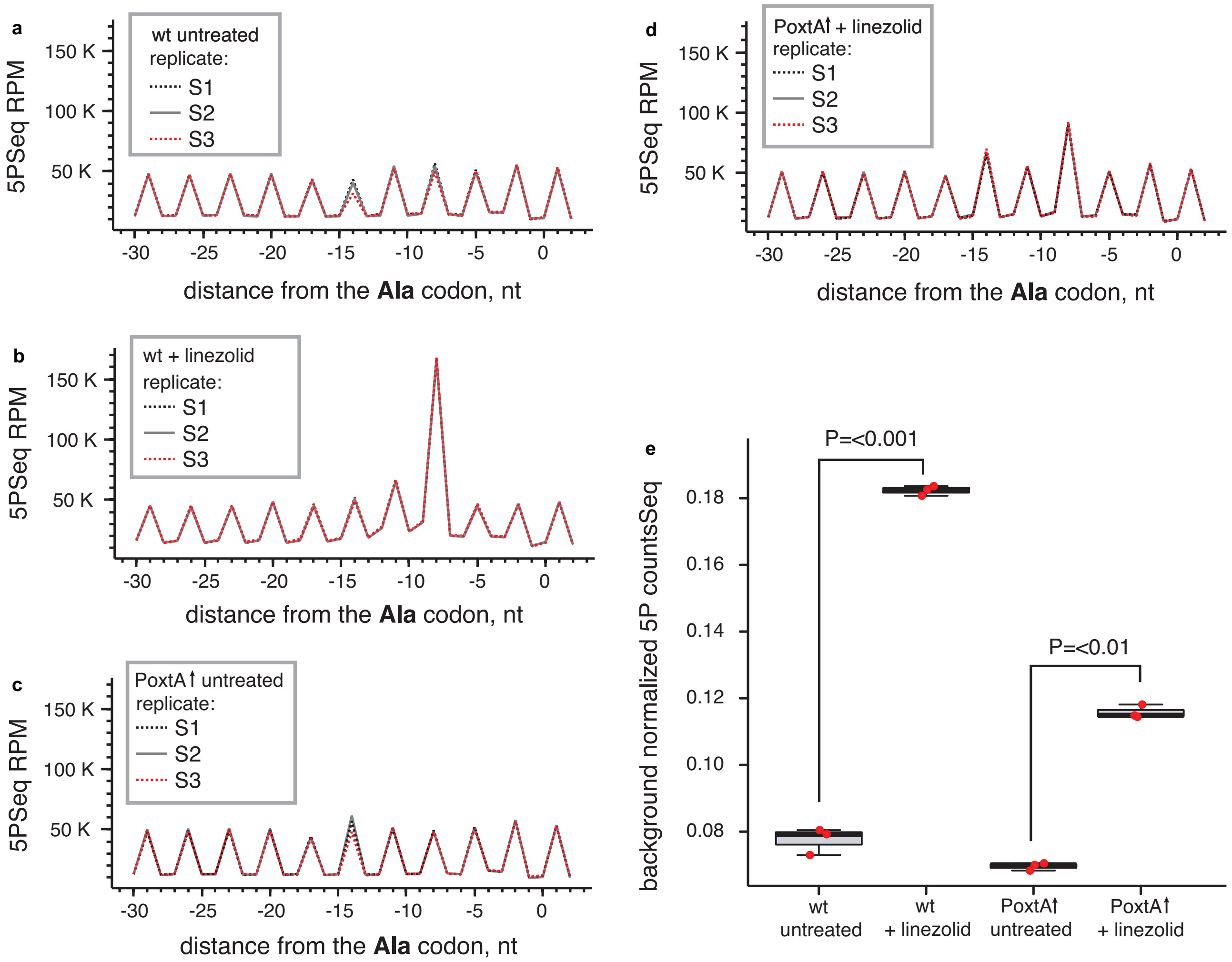

Supplementary Figure 4. 5PSeq data display high reproducibility amongst the three biological replicates. 5PSeq robustly detects ribosomal stalls, with alanine present at the –1 position on the nascent chain, upon treatment with linezolid which are rescued by PoxtA. Metagene plots aligned to alanine, of three biological replicates from either *E. faecalis* harbouring the empty vector pCIE$_{spec}$, without (**a**) or with linezolid treatment (**b**), or expressing *poxtA* without (**c**) or with linezolid treatment (**d**). Note, increased coverage at –8 nt is indicative of a ribosomal stall with the denoted codon in the ribosomal E-site and its decoded amino acid present at position –1 of the nascent chain. **e** Statistical robustness of linezolid-induced alanine stalling and its rescue by PoxtA expression. 5PSeq counts relative to Ala were taken in the range of –30 to –3 nt distance from the first position of the codon and normalised to the sum of counts in that range. These relative counts at –8 nt from Ala were then compared between the groups using a t-test (Data from n=3 biologically independent experiments were used). The whiskers represent the minimum and maximum values of the dataset. The box is bounded by the first (lower 25th percentile) and third (upper 75th percentile) quartile with the center indicating the median (middle value of the dataset, 50th percentile). A two-sided t test was performed to compare the groups. The exact p-values for the WT groups is 0.0001145336, and for the PoxtA groups is 3.597002e-05.

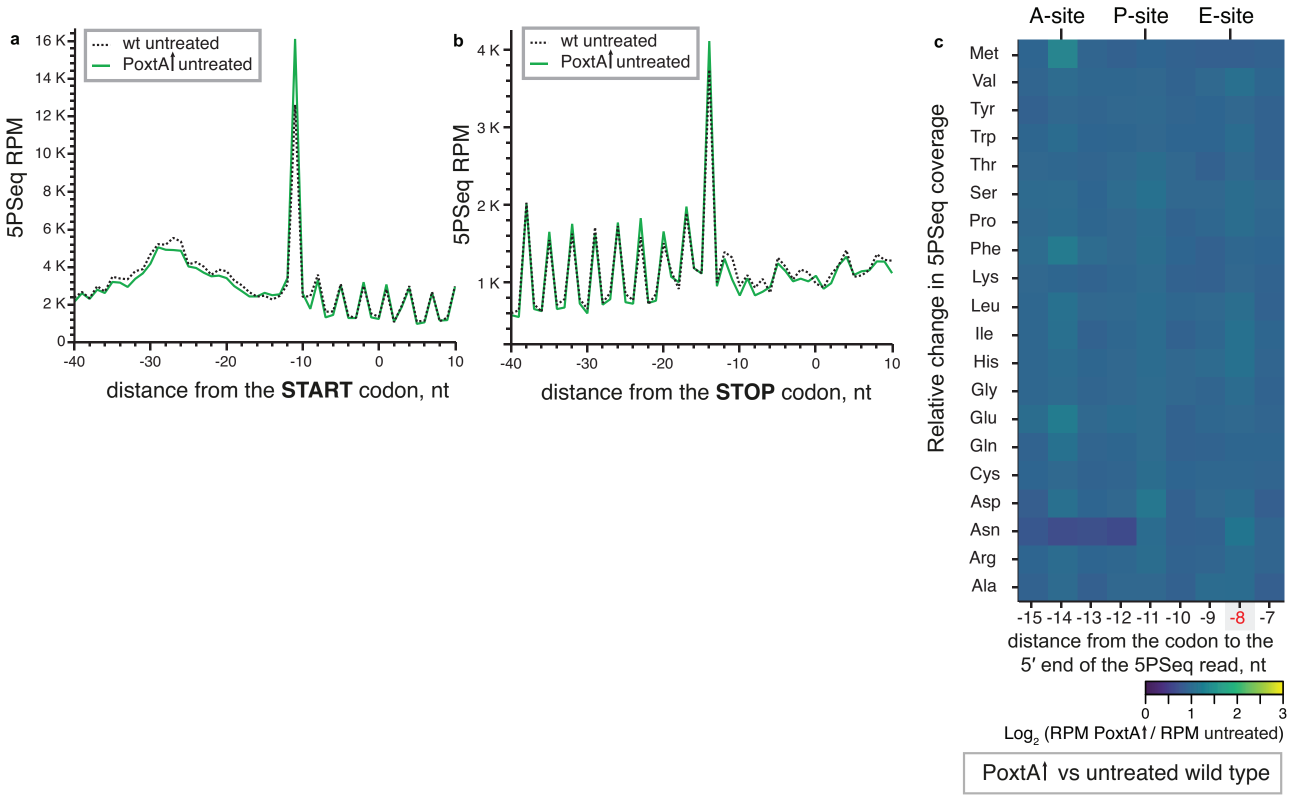

Supplementary Figure 5. PoxtA expression results in context-dependent ribosomal stalling. a, **b** Metagene analysis of 5PSeq coverage in wild-type *E. faecalis* with and without PoxtA expression from the pCIE$_{spec}$ vector, normalised to the first position of the start (**a**) and stop (**b**) codons. (**c**) Heatmaps of relative change in the 5PSeq coverage in relation to specified amino acid codon (or a stop codon) of wild-type *E. faecalis* upon PoxtA expression. The distance (in nucleotides) from the 5' of the sequenced mRNA fragments to indicated codons is indicated on the X axis. Relative change in coverage upon PoxtA expression is colour-coded from dark blue (decreased coverage upon PoxtA expression) to yellow (increased coverage upon PoxtA expression) using the same dynamic range as on **Figure 2a**. Note that the signal from methionine on (**c**) is aggregated from both initiation and elongation codons. All analyses were performed on pooled datasets from three biological replicates.

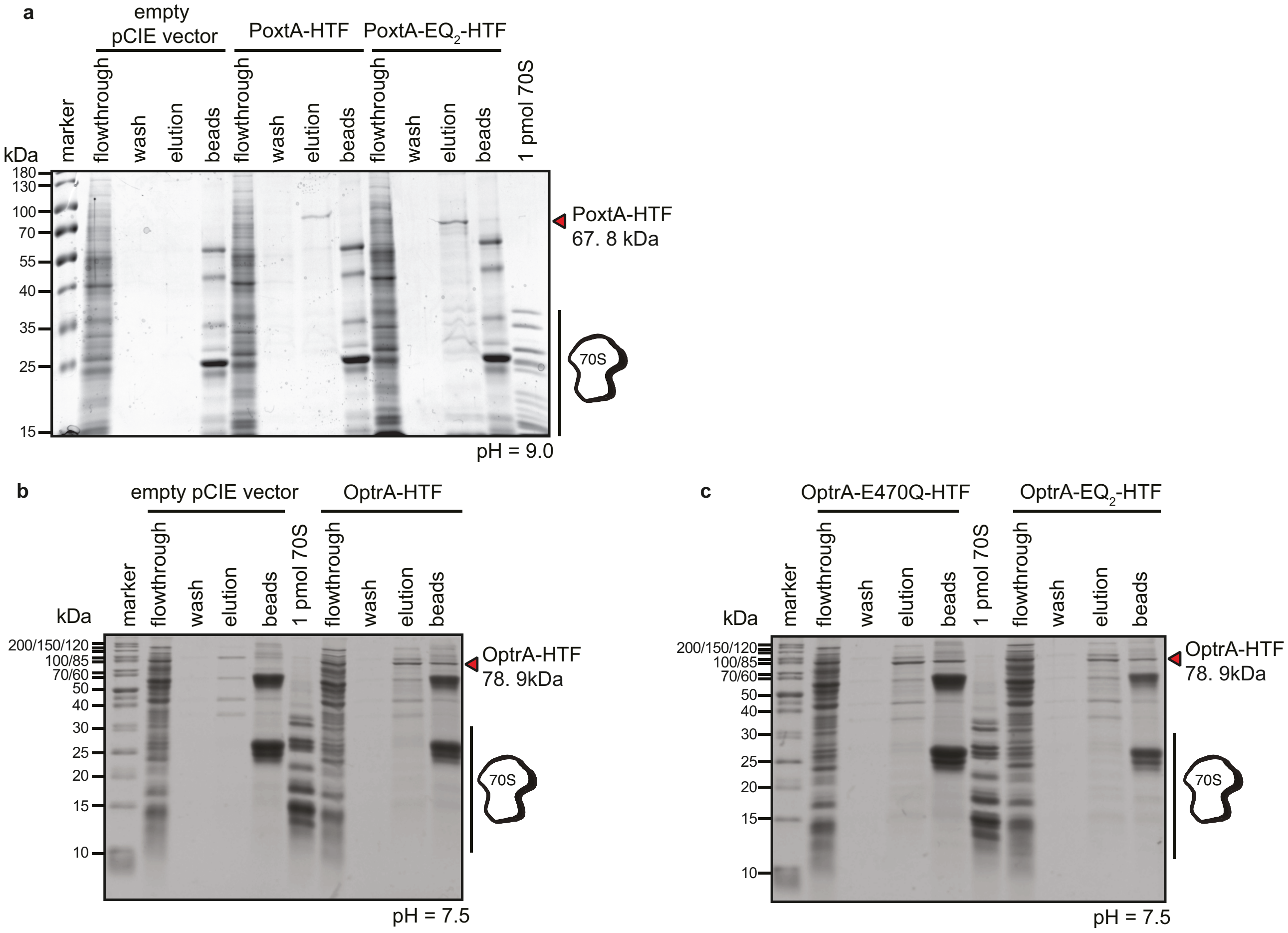

Supplementary Figure 6. Characterization of PoxtA-EF9F6 interactions with ribosomes and preparation of samples for cryo-EM reconstructions. a Affinity purification of PoxtA-EQ$_2$-HTF ectopically expressed in *E. faecalis* Δ*lsaA* (*lsa*::Kan) strain TX5332. Pull-down experiments were performed in the presence of 0.5 mM ATP using clarified lysates of *E. faecalis* either transformed with empty integrative pCIE$_{spec}$ vector (background control), or either expressing PoxtA-HTF or PoxtA-EQ$_2$-HTF. Samples: marker: 2 µL of molecular weight marker; flowthrough: 2 µL of flowthrough; wash: 10 µL of last wash before specific elution; elution: 10 µL of elution with FLAG$_3$ peptide at pH 9.0; beads: 2 µL of SDS-treated post-elution anti-FLAG beads; 70S: purified *E. faecalis* 70S ribosomes, the samples were resolved on 12 % SDS-PAGE gel. **b***,* **c** Affinity purification attempts with wild-type, EQ$_2$ and EQ$_1$ (E470Q) *E. faecalis* OptrA-ST16-HTF ectopically expressed in TX5332 *E. faecalis*. Pull-down experiments were performed in the presence of 0.75 mM ATP at pH 7.5 using clarified lysates of *E. faecalis* either transformed with *E. faecalis* OptrA-ST16-HTF (VHp223) or expressing either *E. faecalis* OptrA-ST16-E470Q-HTF (VHp294) or OptrA-ST16-EQ$_2$-HTF (VHp295). Samples: marker: molecular weight marker; lysate: 2 µL of clarified lysate, flowthrough: 2 µL of flow-through; wash: 10 µL of last wash before specific elution; elution: 10 µL of the elution with FLAG$_3$ peptide; B: 10 µL of SDS-treated post-elution anti-FLAG beads; 70S: purified *E. faecalis* 70S ribosomes. The samples were resolved on 15% SDS-PAGE gel. Each experiment was repeated independently twice with similar results and representative gels are shown.

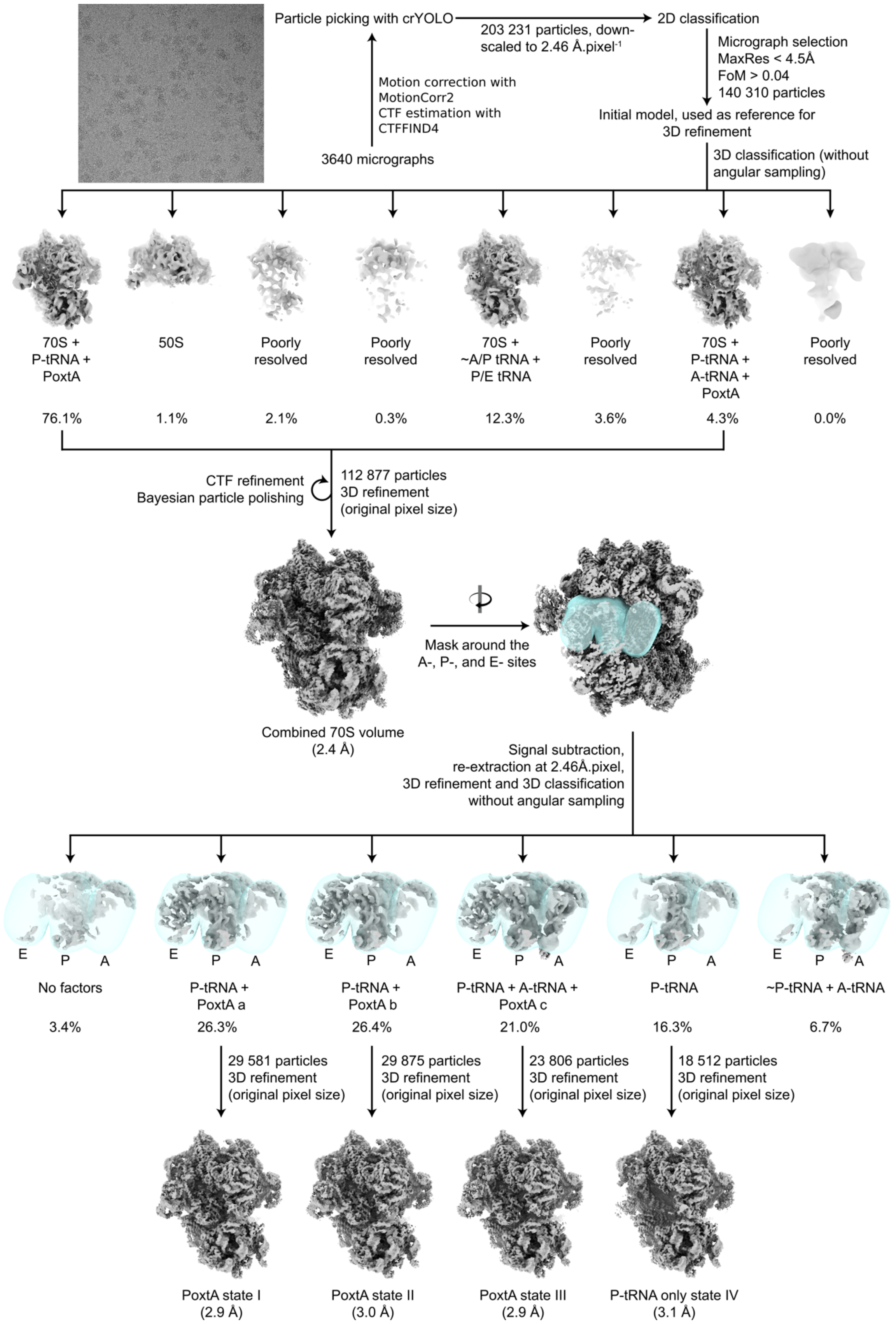

Supplementary Figure 7. Processing scheme for the PoxtA-70S complex. A representative micrograph (low-pass filtered to 15Å) is shown top left. All steps were performed with RELION unless otherwise specified. See also Methods for details.

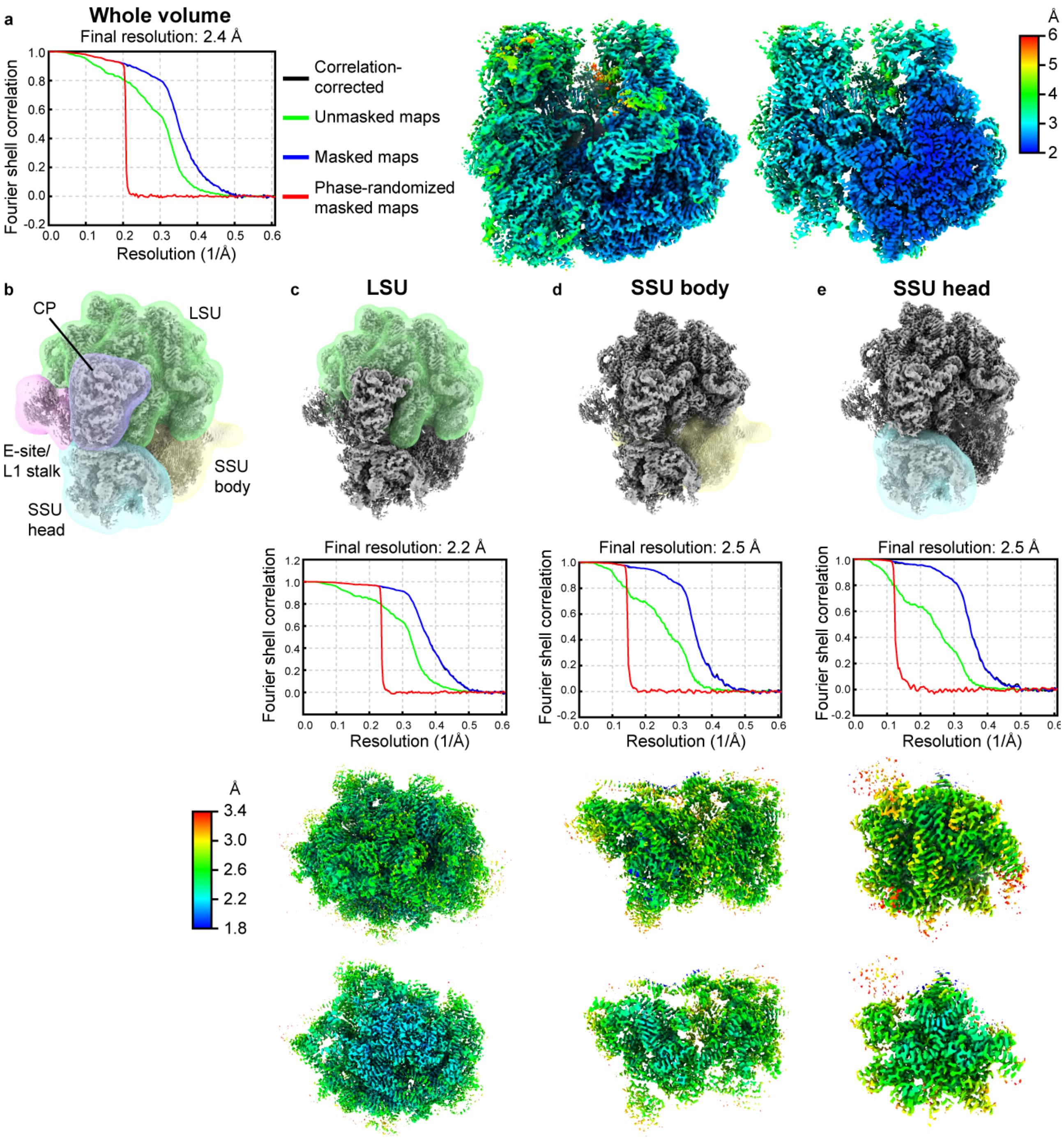

Supplementary Figure 8. Local resolutions and multibody refinements for the combined 70S volume. a FSC curve and local resolution images for the combined 70S volume. **b** Overview of masks used for multibody refinement (**c**–**e** masks relative to whole 70S (top row), FSC curves (second row), and density coloured according to local resolution (bottom two rows) for (**c**) the LSU core, (**d**) the SSU body, and (**e**) the SSU head.

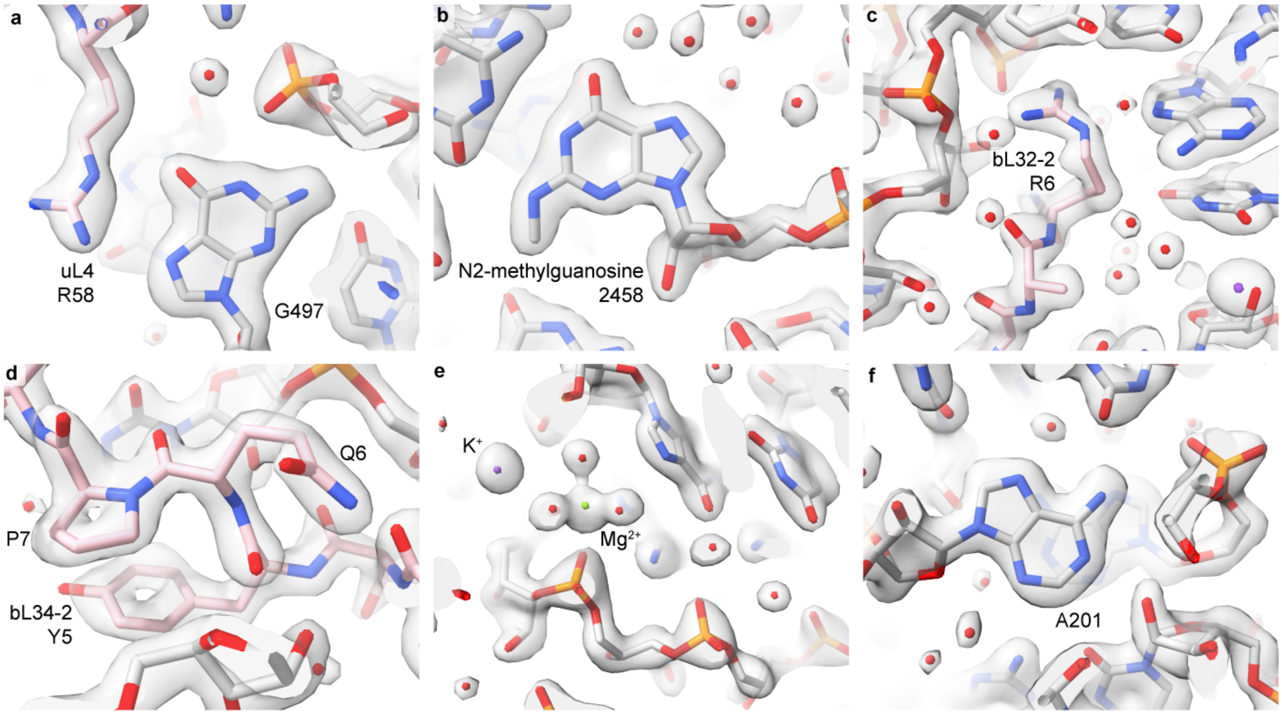

Supplementary Figure 9. Selected density images from the high-resolution LSU core. Modelled water molecules are colored red, magnesium green, and potassium purple. **a** Interaction between uL4 and 23S rRNA. **b** N2-methylguanosine in 23S rRNA. **c** Interaction between bL32-2 and 23S rRNA. **d** A portion of bL34-2. **e** Focus on modelled solvent in the LSU. **f** A portion of the 23S rRNA showing the clearly resolved N6 of A201.

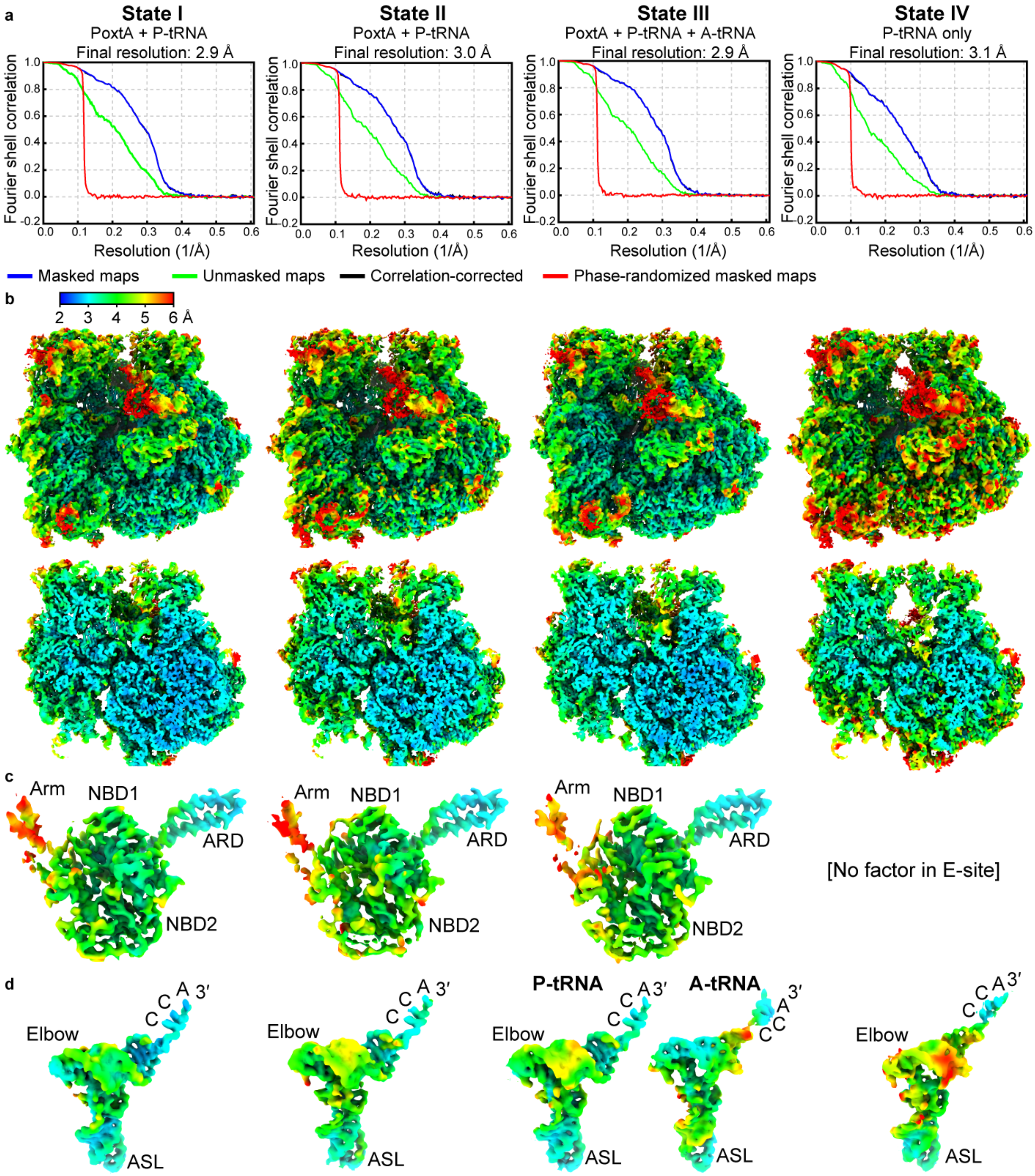

Supplementary Figure 10. FSC curves and density coloured by local resolution for states I-IV. a FSC curves. **b** density coloured by local resolution for whole volumes with cut-throughs (bottom row). **c** Isolated density for isolated PoxtA coloured by local resolution. **d** Isolated density for isolated tRNAs coloured by local resolution.

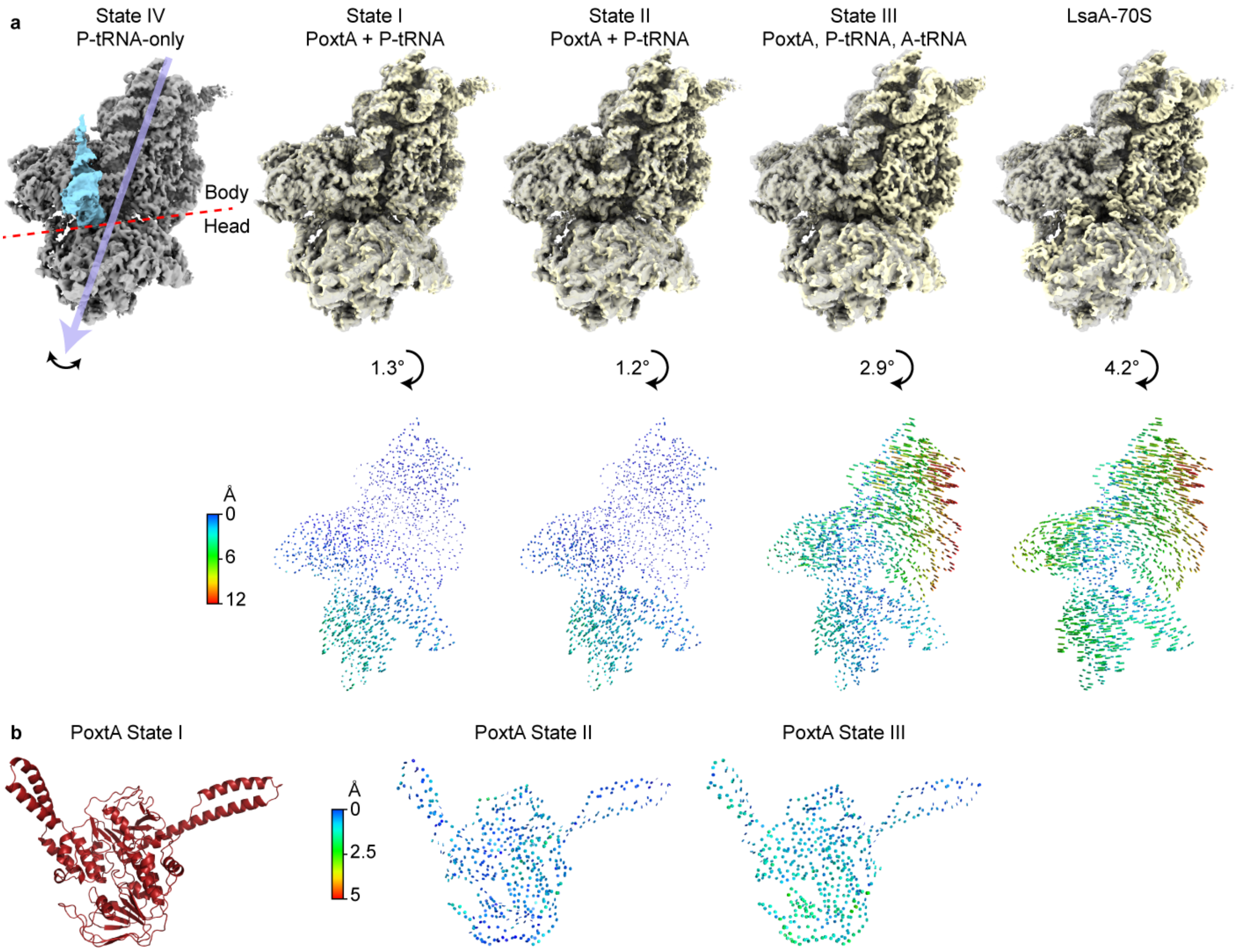

Supplementary Figure 11. Comparison of subunit rotation and PoxtA among states I-IV. a Rotation of the small subunit. The SSU with P-tRNA from state IV (P-tRNA-only) is shown on the left. A dotted red line demarcates the boundary between the head and body. The transparent blue line indicates the major direction of rotation of the whole subunit, which can move left to right in this representation. The arrow below indicates the extent of measured rotation. For comparisons, cryo-EM density of states I–III or the LsaA–70S volume (EMD-12331)[15] are shown in yellow, while the state IV density is in transparent grey. In the row below, difference vectors for P atoms drawn around the rotation axis and coloured according to distance are shown. LsaA–70S, PDB ID 7NHK[15]. **b** Comparison of PoxtA between states I, II and III. Difference vectors for the C_α atoms are shown between state I and states II and III.

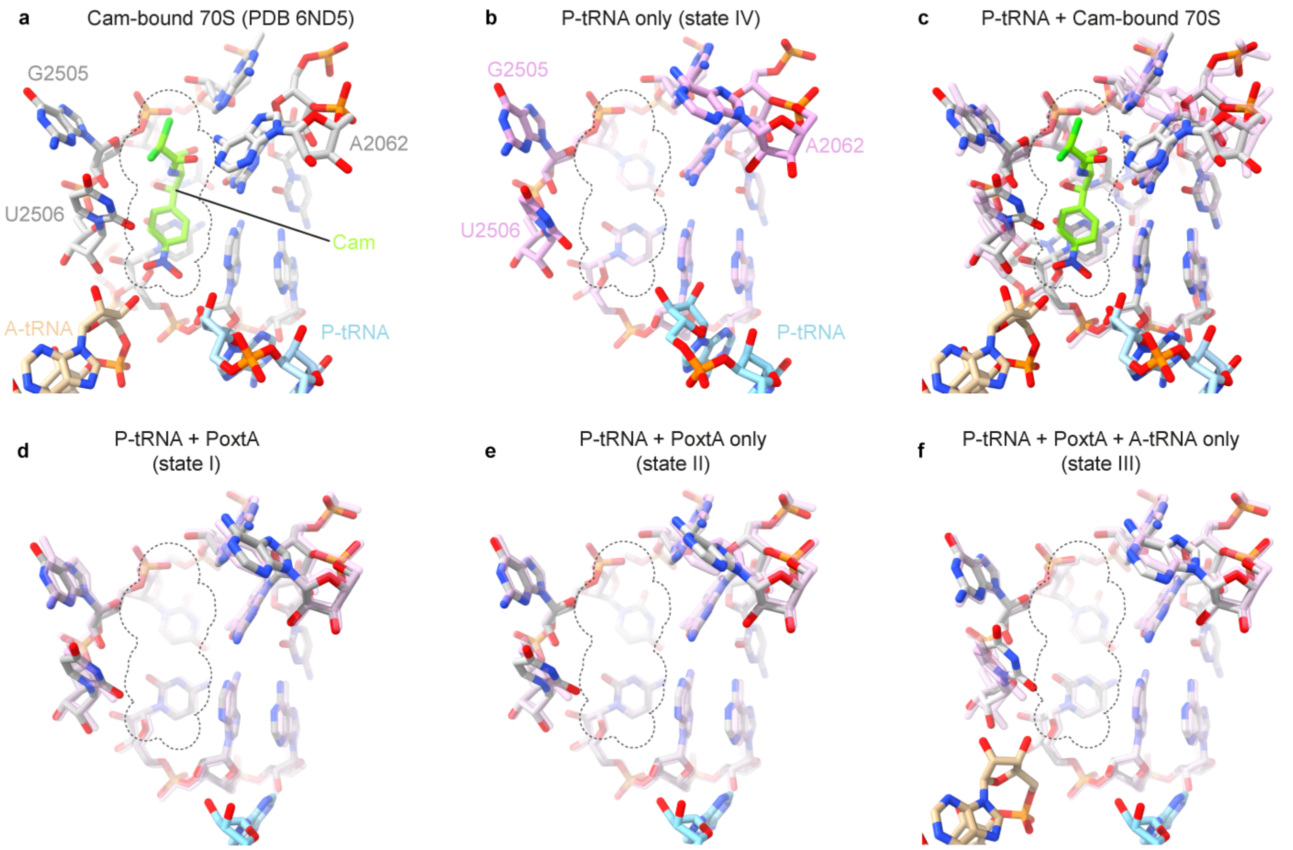

Supplementary Figure 12. Effects of PoxtA binding on the 23S rRNA chloramphenicol binding site. a Chloramphenicol (cam, green, PDB ID 6ND5[78]) bound to 23S rRNA (grey). Dotted lines indicate the extent of a space-filling model of chloramphenicol. A-tRNA (tan) and P-tRNA (light blue) are also shown. **b** Same view as **a** but for the P-tRNA-only bound volume (state IV). 23S rRNA is shown in pink. **c** Same as panel A but with P-tRNA-only (state IV) 23S rRNA superimposed (transparent pink). **d**–**f** As for **c**, but comparing the P-tRNA-only (state IV) 23S rRNA with PoxtA bound states I–III. The chloramphenicol outline is shown for reference.

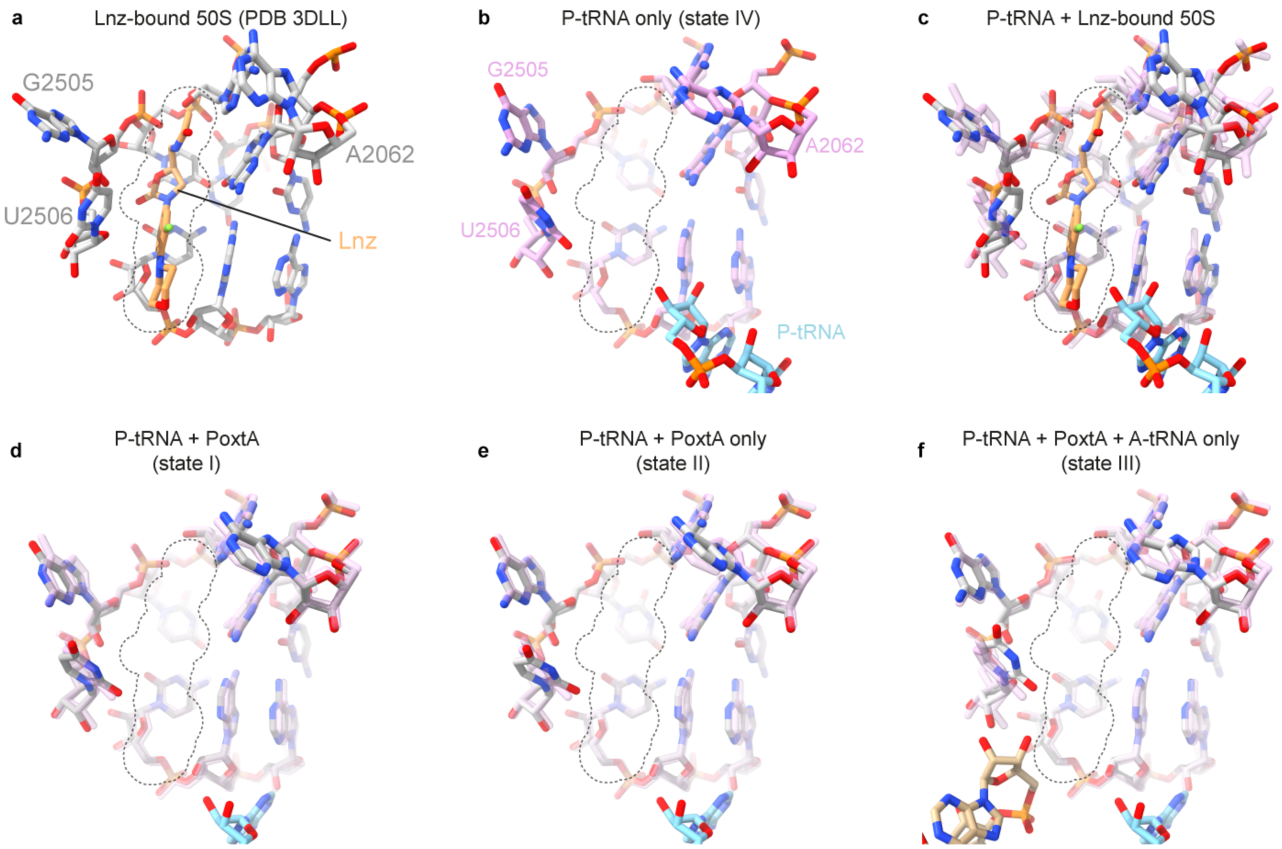

Supplementary Figure 13. Effects of PoxtA binding on the 23S rRNA linezolid binding site. a Linezolid (Lnz, light orange, PDB ID 3DLL[80]) bound to 23S rRNA (grey). Dotted lines indicate the extent of a space-filling model of linezolid. **b** Same view as **a** but for the P-tRNA-only bound volume (state IV). 23S rRNA is shown in pink. **c** Same as panel **a** but with P-tRNA-only (state IV) 23S rRNA superimposed (transparent pink). **d**–**f** As for **c**, but comparing the P-tRNA-only (state IV) 23S rRNA with PoxtA bound states I–III. The linezolid outline is shown for reference.
