# Supplementary material for: Structural basis for PoxtA-mediated resistance to phenicol and oxazolidinone antibiotics
Source: Nat Commun. 2022 Apr 6;13:1860. doi: 10.1038/s41467-022-29274-9 (PMC8987054; doi:10.1038/s41467-022-29274-9)
Supplement: Supplementary file 2 — Reporting Summary [file 41467_2022_29274_MOESM2_ESM.pdf]

nature portfolio

Corresponding author(s): Wilson DN
Hauryliuk V

Last updated by author(s): Feb 14, 2022

Reporting Summary

Nature Portfolio wishes to improve the reproducibility of the work that we publish. This form provides structure for consistency and transparency in reporting. For further information on Nature Portfolio policies, see our Editorial Policies and the Editorial Policy Checklist.

Statistics

For all statistical analyses, confirm that the following items are present in the figure legend, table legend, main text, or Methods section.

n/a	Confirmed	
☐	☒	The exact sample size (*n*) for each experimental group/condition, given as a discrete number and unit of measurement
☐	☒	A statement on whether measurements were taken from distinct samples or whether the same sample was measured repeatedly
☐	☒	The statistical test(s) used AND whether they are one- or two-sided<br>*Only common tests should be described solely by name; describe more complex techniques in the Methods section.*
☒	☐	A description of all covariates tested
☒	☐	A description of any assumptions or corrections, such as tests of normality and adjustment for multiple comparisons
☐	☒	A full description of the statistical parameters including central tendency (e.g. means) or other basic estimates (e.g. regression coefficient) AND variation (e.g. standard deviation) or associated estimates of uncertainty (e.g. confidence intervals)
☐	☒	For null hypothesis testing, the test statistic (e.g. *F*, *t*, *r*) with confidence intervals, effect sizes, degrees of freedom and *P* value noted<br>*Give P values as exact values whenever suitable.*
☒	☐	For Bayesian analysis, information on the choice of priors and Markov chain Monte Carlo settings
☒	☐	For hierarchical and complex designs, identification of the appropriate level for tests and full reporting of outcomes
☒	☐	Estimates of effect sizes (e.g. Cohen's *d*, Pearson's *r*), indicating how they were calculated

Our web collection on statistics for biologists contains articles on many of the points above.

Software and code

Policy information about availability of computer code

Data collection	CryoEM data were collected using the EPU 2.6.1 software (FEI, Netherlands)
Data analysis	RELION v3.1 with MotionCor2 v1.2.1, CTFFIND 4, and crYOLO v1.6.1 were used for processing micrographs, picking particles, classification and refining cryo-EM maps. BSoft 2.1.1 was used to calculate local resolution. Coot v0.9.5 EL and v0.9-pre EL and ISOLDE v1.1.0 for model building and Phenix (1.19.2 and later development versions) for model refinement and statistics. Figures were generated using Pymol v2.4 and ChimeraX v1.1 and v1.2.

For manuscripts utilizing custom algorithms or software that are central to the research but not yet described in published literature, software must be made available to editors and reviewers. We strongly encourage code deposition in a community repository (e.g. GitHub). See the Nature Portfolio guidelines for submitting code & software for further information.

Data

Policy information about availability of data

All manuscripts must include a data availability statement. This statement should provide the following information, where applicable:

- Accession codes, unique identifiers, or web links for publicly available datasets
- A description of any restrictions on data availability
- For clinical datasets or third party data, please ensure that the statement adheres to our policy

Micrographs have been deposited as uncorrected frames in the Electron Microscopy Public Image Archive (EMPIAR) with the accession codes EMPIAR-10764 [https://www.ebi.ac.uk/pdbe/emdb/empiar/entry/10764/]. Cryo-EM maps have been deposited in the Electron Microscopy Data Bank (EMDB) with accession codes EMD-13241 [https://www.ebi.ac.uk/pdbe/entry/emdb/EMD-13241] (E. faecalis combined 70S volume), EMD-13242 [https://www.ebi.ac.uk/pdbe/entry/emdb/EMD-13242] (PoxtA-70S state I), EMD-13243 [https://www.ebi.ac.uk/pdbe/entry/emdb/EMD-13243] (PoxtA-70S state II), EMD-13244 [https://www.ebi.ac.uk/pdbe/

entry/emdb/EMD-13244] (PoxtA-70S state III with A-site tRNA) and EMD-13245 [https://www.ebi.ac.uk/pdbe/entry/emdb/EMD-13245] (E. faecalis 70S with P-tRNA state IV). Molecular models have been deposited in the Protein Data Bank with accession codes 7P7Q [https://doi.org/10.2210/pdb7P7Q/pdb] (E. faecalis combined 70S volume), 7P7R [https://doi.org/10.2210/pdb7P7R/pdb] (PoxtA-70S state I) 7P7S [https://doi.org/10.2210/pdb7P7S/pdb] (PoxtA-70S state II), 7P7T [https://doi.org/10.2210/pdb7P7T/pdb] (PoxtA-70S state III with A-site tRNA) and 7P7U [https://doi.org/10.2210/pdb7P7U/pdb] (E. faecalis 70S with P-tRNA, state IV). Source data are provided with this paper. Sequencing data are deposited at GEO with accession code GSE179348 [https://www.ncbi.nlm.nih.gov/geo/query/acc.cgi?acc=GSE179348].

Field-specific reporting

Please select the one below that is the best fit for your research. If you are not sure, read the appropriate sections before making your selection.

☒ Life sciences ☐ Behavioural & social sciences ☐ Ecological, evolutionary & environmental sciences

For a reference copy of the document with all sections, see nature.com/documents/nr-reporting-summary-flat.pdf

Life sciences study design

All studies must disclose on these points even when the disclosure is negative.

Sample size	MIC experiments were done in triplicates using three individual bacterial cultures, and 5PSeq experiments were performed in triplicates, as per convention.
Data exclusions	Micrographs with low estimated resolution or poorly fitted CTFs were discarded, as were particles that clustered into poorly defined classes during 2D and 3D classification.
Replication	For MIC and 5PSeq experiments individual independent cultures were used to generate independent biological replicates. For the 5Pseq the triplicates were successfully performed as shown in Supplementary Figure 2. MIC triplicates were successful and presented in Table S1.
Randomization	For 3D refinement in RELION, particles are randomly placed in one of two subsets. These subsets are maintained for CTF refinement. Otherwise, no randomization was performed.
Blinding	No blinding was performed as blinding is not possible or not applicable for the experiments.

Reporting for specific materials, systems and methods

We require information from authors about some types of materials, experimental systems and methods used in many studies. Here, indicate whether each material, system or method listed is relevant to your study. If you are not sure if a list item applies to your research, read the appropriate section before selecting a response.

Materials & experimental systems

n/a	Involved in the study
☒	☐ Antibodies
☒	☐ Eukaryotic cell lines
☒	☐ Palaeontology and archaeology
☒	☐ Animals and other organisms
☒	☐ Human research participants
☒	☐ Clinical data
☒	☐ Dual use research of concern

Methods

n/a	Involved in the study
☒	☐ ChIP-seq
☒	☐ Flow cytometry
☒	☐ MRI-based neuroimaging
